# Transcriptional Profiling of Monocytes Deficient in Nuclear Orphan Receptors *NR4A2* and *NR4A3* Reveals Distinct Signalling Roles Related to Antigen Presentation and Viral Response

**DOI:** 10.3389/fimmu.2021.676644

**Published:** 2021-06-25

**Authors:** David E. Phelan, Masahiko Shigemura, Sarah Aldhafiri, Catarina Mota, Thomas J. Hall, Jacob I. Sznajder, Evelyn P. Murphy, Daniel Crean, Eoin P. Cummins

**Affiliations:** ^1^ School of Medicine, University College Dublin, Dublin, Ireland; ^2^ Conway Institute of Biomolecular and Biomedical Research, University College Dublin, Dublin, Ireland; ^3^ Division of Pulmonary and Critical Care Medicine, Feinberg School of Medicine, Northwestern University, Chicago, IL, United States; ^4^ Animal Genomics Laboratory, School of Veterinary Medicine, University College Dublin, Dublin, Ireland; ^5^ School of Agriculture and Food Science, University College Dublin, Dublin, Ireland; ^6^ School of Medicine, University of Limerick, Limerick, Ireland

**Keywords:** NR4A, NR4A2, NR4A3, nuclear orphan receptor, nuclear receptor, monocytes, cell signalling, transcriptomics

## Abstract

The nuclear receptor sub-family 4 group A (NR4A) family are early response genes that encode proteins that are activated in several tissues/cells in response to a variety of stressors. The NR4A family comprises *NR4A1*, *NR4A2* and *NR4A3* of which *NR4A2* and *NR4A3* are under researched and less understood, particularly in the context of immune cells. NR4A expression is associated with multiple diseases e.g. arthritis and atherosclerosis and the development of NR4A-targetting molecules as therapeutics is a current focus in this research field. Here, we use a combination of RNA-sequencing coupled with strategic bioinformatic analysis to investigate the down-stream effects of *NR4A2* and *NR4A3* in monocytes and dissect their common and distinct signalling roles. Our data reveals that *NR4A2* and *NR4A3* depletion has a robust and broad-reaching effect on transcription in both the unstimulated state and in the presence of LPS. Interestingly, many of the genes affected were present in both the unstimulated and stimulated states revealing a previously unappreciated role for the NR4As in unstimulated cells. Strategic clustering and bioinformatic analysis identified both distinct and common transcriptional roles for *NR4A2* and *NR4A3* in monocytes. *NR4A2* notably was linked by both bioinformatic clustering analysis and transcription factor interactome analysis to pathways associated with antigen presentation and regulation of MHC genes. *NR4A3* in contrast was more closely linked to pathways associated with viral response. Functional studies further support our data analysis pointing towards preferential/selective roles for *NR4A2* in the regulation of antigen processing with common roles for *NR4A2* and *NR4A3* evident with respect to cell migration. Taken together this study provides novel mechanistic insights into the role of the enigmatic nuclear receptors NR4A2 and NR4A3 in monocytes.

## Introduction

The nuclear receptor sub-family 4 group A (NR4A) family are early response genes that are activated in response to a variety of stimuli and stressors (1, 2). There are 3 genes in this family: *NR4A1* (*Nur77*, *TR3*, *NGFI-B*), *NR4A2* (*Nurr1*), and *NR4A3* (*Nor1*). They have relatively wide tissue specificity, with generally low RNA levels and varying levels of protein detected in a wide range of tissues/cells. Of the 3 family members, *NR4A1* is perhaps the most widely studied followed by *NR4A2*, with *NR4A3* being the least investigated. NR4A expression is associated with a number of pathologies e.g. arthritis (3–6), T cell dysfunction (7), fibrosis (8), atherosclerosis (9) and cancer (10, 11). Furthermore, over the last decade NR4A receptors have become targets for pharmacological intervention. This approach is in its infancy, but the role of these agonists/antagonists is consistently developing (12, 13).

There is a high degree of sequence homology and structural similarity between the different NR4A family members. Structural elements include a ligand-independent activation-function 1 (AF-1) transactivation domain in the N-terminal region, a zinc finger containing DNA-binding domain, a ligand binding domain, and a ligand-dependent AF-1 domain (13). The ligand binding domain of the NR4As is different to that of other nuclear receptors with tightly packed hydrophobic residues present instead of traditional ligand-binding domain (LBD) of other nuclear receptors. These relatively inaccessible ligand binding pockets led to the NR4As being described as nuclear orphan receptors due to the absence of a known endogenous ligand. More recently however, de Vera et al. have established that the LBD of NR4A2 is dynamic and can exchange between conformations allowing the binding of unsaturated fatty acids (14). Additionally, a range of structurally diverse synthetic compounds have been identified as interacting with the ligand binding domain of NR4A1 acting as agonists or antagonists. Additional ligands have been discovered for NR4A2 and NR4A3 (15). In the absence of endogenous ligands, the most common mechanisms through which the transcriptional activity of NR4A family members are known to be regulated are changes in mRNA levels and post-translational modifications, specifically phosphorylation and SUMOylation (16–19). The NR4As are known to function as transcriptional regulators by binding directly to specific DNA sequences in the promoter regions of target genes. In addition, the NR4As play a role as co-factors by forming transcriptional regulatory complexes with other proteins to modulate gene expression without directly binding DNA. As monomers, the NR4As bind to the NGFI-B response element (NBRE) sequence (AAAGGTCA) (13). As homodimers or NR4A1/NR4A2 – heterodimers (not NR4A3) the NR4As can additionally bind to the palindromic Nur-response element (TGATATTTACCTC CAAAATCCA) (20–22). NR4A1 and NR4A2 (but not NR4A3) can additionally heterodimerise with retinoid X receptors to bind DR5 elements. Taken together the NR4As can affect transcription directly *via* binding DNA as monomers, homodimers, and heterodimers as well as through co-factor activity with other transcriptional regulators to modulate the expression of genes involved in key cellular functions including inflammation, cell survival and proliferation (23).

Given the sequence and structural similarity of NR4A family members one would anticipate a strong degree of concordance between genes regulated by the NR4A. While there is evidence that this is the case for certain genes in specific cell types, there is also evidence to suggest that individual NR4A family members have unique signalling roles. The relative contribution of individual NR4A family members to gene expression is influenced by the relative expression and subcellular localisation of NR4A family members in individual cell and tissue sub-types but is also likely influenced by the physiological function and local microenvironment of that cell or tissue. NR4A1-3 stimulus- and cell context-dependent activities are further due, in part, to their selective interaction with other factors. Review of the NR4A1-3 interactome highlights that, while each family member interacts with a wide range of factors, they share a limited number of common factors thus contributing to unique cellular functions (24, 25). Specifically, NR4A2 and NR4A3 have been implicated in the regulation of inflammatory signalling in numerous studies (26–31). However, to date unbiased transcriptomic approaches have yet to be performed on myeloid cells in order to directly compare and contrast what pathways are regulated by NR4A2 and NR4A3 in both the stimulated and unstimulated state. In this study we use a combination of RNA-sequencing coupled with strategic bioinformatic analysis to investigate the down-stream effects of NR4A2 and NR4A3 depletion in monocytes and dissect their common and distinct signalling roles in these cells.

## Materials and Methods

### Cell Culture

shNT, shNR4A2 and shNR4A3 THP-1 cells previously generated (28) were maintained in RPMI 1640 + Glutamax, (supplemented with 10% FBS, 1% P/S, and 2.5μg/ml puromycin) in a humidified incubator (37°C, 5% CO_2_). Experiments were performed in RPMI media as above or buffered DMEM media (Per 500ml media: 445ml DMEM solution (Sigma Aldrich D1152), 5ml P/S, 50ml FCS, 1.23g NaHCO_3_, 0.71g NaCl) supplemented with 10% FBS, 1% P/S.

### RNA-Seq

The appropriate number of shNT, shNR4A2 and shNR4A3 THP-1 cells were counted, centrifuged at 1,200rpm (290xg) and the supernatant was removed. Cells were transferred to an environmental gas chamber (37°C, 5% CO_2_) and resuspended in buffered DMEM media supplemented with 10% FBS, 1% P/S. at a concentration of 750,000 cells/ml. Cells were incubated for 2 hours. LPS-stimulated cells were treated with 2.5μg/ml LPS (Invivogen) for a further 2 hours. After a total of 4 hours, all cells were collected and total mRNA was extracted using the Omega EZNA Total RNA Kit I, as per the manufacturer’s instructions.

**Figure 1:**
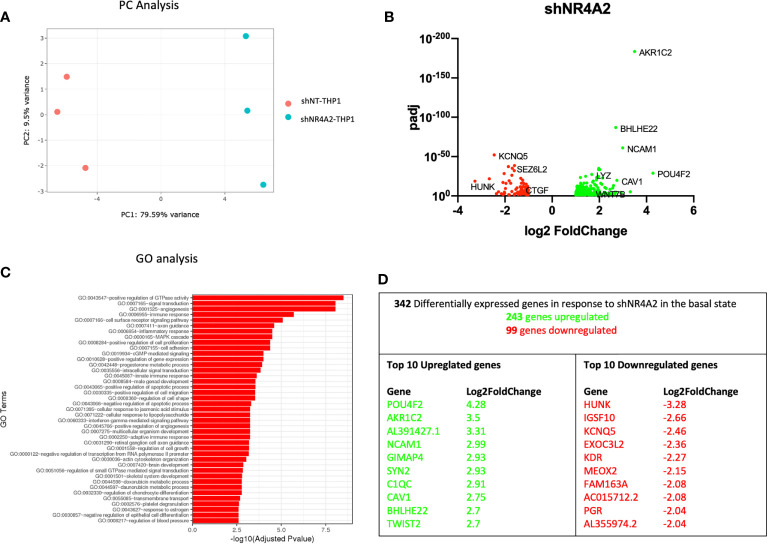
Knockdown of *NR4A2* has a distinct transcriptional profile in the basal state. Principal component analysis (PCA) plot of transduced cells based on RNA-seq data between shNT and shNR4A2 cells **(A)**. Volcano plot of significant DEGs (p<0.05) upregulated (log2fc>/=1) (green) or downregulated (log2fc</=-1) (red) by knockdown of *NR4A2* in THP-1 cells based on RNA-seq data **(B)**. Top gene ontology (GO) terms associated with knockdown of *NR4A2* in THP-1 cells based on RNA-seq data **(C)**. Summary table of significant DEGs upregulated (green) and downregulated (red) by knockdown of *NR4A2* in THP-1 cells based on RNA-seq data **(D)**.

RNA quality was assessed by bioanalyser (Agilent Technologies) ensuring a RIN number of >8. Qubit results also ensured a RIN score >9.5 for all samples. cDNA library preparation was performed with polyA selection using Illumina HiSeq, 2x150bp configuration, single index, per lane. ~350M raw paired-end reads per lane. Library preparation and sequencing were performed by Genewiz (Germany). Sequencing data was analysed using DESeq2 (32). Initial GO analysis was performed using GeneSCF v1.1-p2 to generate GO graphs where a Fisher Exact test was used to determine p-values (**Figures 1** **–** **4**).

**Figure 2:**
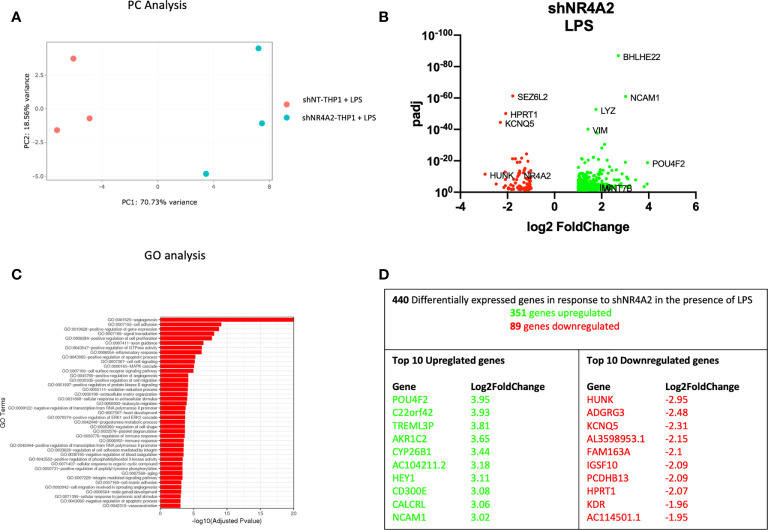
Knockdown of *NR4A2* has a distinct transcriptional profile in the presence of LPS. Principal component analysis (PCA) plot of transduced cells based on RNA-seq data between shNT and shNR4A2 cells in the presence of LPS **(A)**. Volcano plot of significant DEGs (p<0.05) upregulated (log2fc>/=1) (green) or downregulated (log2fc</=-1) (red) by knockdown of *NR4A2* in THP-1 cells in the presence of LPS based on RNA-seq data **(B)**. Top gene ontology (GO) terms associated with knockdown of *NR4A2* in THP-1 cells in the presence of LPS based on RNA-seq data **(C)**. Summary table of significant DEGs upregulated (green) and downregulated (red) by knockdown of *NR4A2* in THP-1 cells in the presence of LPS based on RNA-seq data **(D)**.

**Figure 3:**
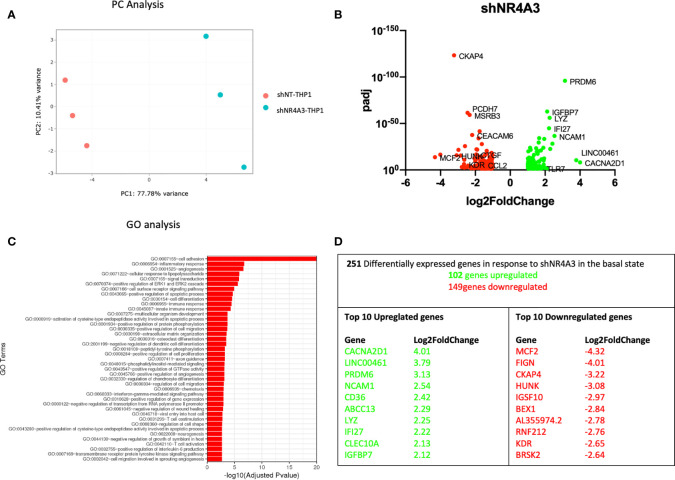
Knockdown of *NR4A3* has a distinct transcriptional profile in the basal state. Principal component analysis (PCA) plot of transduced cells based on RNA-seq data between shNT and shNR4A3 cells **(A)**. Volcano plot of significant DEGs (p<0.05) upregulated (log2fc>/=1) (green) or downregulated (log2fc</=-1) (red) by knockdown of *NR4A3* in THP-1 cells based on RNA-seq data **(B)**. Top gene ontology (GO) terms associated with knockdown of *NR4A3* in THP-1 cells based on RNA-seq data **(C)**. Summary table of significant DEGs upregulated (green) and downregulated (red) by knockdown of *NR4A3* in THP-1 cells based on RNA-seq data **(D)**.

**Figure 4:**
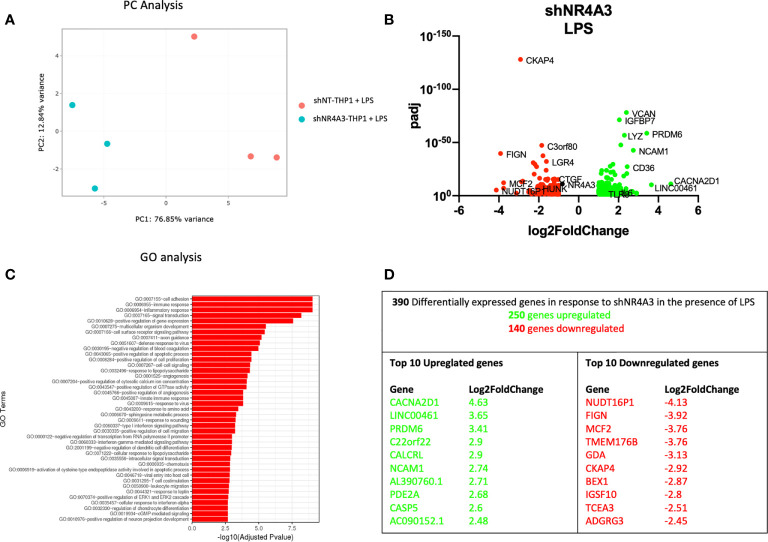
Knockdown of *NR4A3* has a distinct transcriptional profile in the presence of LPS. Principal component analysis (PCA) plot of transduced cells based on RNA-seq data between shNT and shNR4A3 in the presence of LPS cells **(A)**. Volcano plot of significant DEGs (p<0.05) upregulated (log2fc>/=1) (green) or downregulated (log2fc</=-1) (red) by knockdown of NR4A3 in THP-1 cells in the presence of LPS based on RNA-seq data **(B)**. Top gene ontology (GO) terms associated with knockdown of *NR4A3* in THP-1 cells in the presence of LPS based on RNA-seq data **(C)**. Summary table of significant DEGs upregulated (green) and downregulated (red) by knockdown of *NR4A3* in THP-1 cells in the presence of LPS based on RNA-seq data **(D)**.

### RNA-Seq Data Analysis

Significant differentially expressed genes (DEGs) were determined as any genes from the deseq2 workflow, which had an adjusted p -value (padj) <0.05 (**Supplementary Data Tables 1**–**4**). To compare across multiple groups and identify sub clusters of shNR4A2 and shNR4A3 specific genes, nested comparisons were performed. Nested comparisons were completed by cross referencing lists of DEGs from pairwise comparisons to generate secondary lists of either common or specific DEGs. Gene ontology (GO) analysis was performed on each list of DEGs (resulting from pairwise and nested comparisons) (**Supplementary Data Tables 11**–**13**). This analysis was completed by the PANTHER Classification system (pantherdb.org) using statistical overrepresentation tests. The background reference list for these comparisons was generated by compilation of all genes expressed to any degree in any of the experimental conditions (raw TPM values). Raw data from the RNA-seq experiment is displayed as mean TPM (+/-SEM) in order to best visualise expression changes between several groups. Statistical comparisons between individual groups used DESeq2 analysis of normalised counts. The adjusted p-values for specific comparisons are shown in tabular form beneath the TPM data in main figures and **Supplementary Data**. Where an adjusted p-value is not available it is denoted N/A, when an adjusted p-value could not be calculated it is denoted as NA.

### Western Blot

The appropriate number of THP-1 cells were counted, centrifuged at 1,200rpm (290xg) and the supernatant was removed. Cells were resuspended in RPMI media supplemented with 10% FBS, 1% P/S. Cells were incubated for 2 hours in a humidified incubator (37°C, 5% CO_2_) before being stimulated +/- 2.5μg/ml LPS (Invivogen) for up to 24 hours. After a total of 26 hours, all cells were collected, lysed in whole cell lysis buffer (0.02M Tris, 0.15M NaCl, 0.001M MgCl_2_, 1% Triton-X). Protein was quantified by DC Protein Assay (Bio-Rad) and electrophoresed on 8% SDS PAGE gel followed by transfer to nitrocellulose membranes (GE Healthcare). Protein expression levels were measured by Western blot analysis using specific antibodies for NR4A2 (Invitrogen MA1-195) and β-actin (Sigma A5441) Briefly, nitrocellulose membranes were blocked in blocking buffer (1X Tris Buffered Saline with Tween-20 (0.1%) (TBST) containing 5% w/v skimmed milk) and incubated with primary antibodies overnight at 4°C in blocking buffer. Nitrocellulose membranes were washed 5 times for 10 min with 1× TBST and subsequently incubated for 1 h with HRP- conjugated anti-mouse secondary antibodies (Cell Signaling Technology, 7076S) diluted in blocking buffer at room temperature. Membranes were washed again as described followed by signal detection using an enhanced chemiluminescent substrate (Thermo Scientific, 32106) followed by exposure of the membrane to x-ray films in a dark room.

### Quantitative Polymerase Chain Reaction (qPCR)

The appropriate number of wild-type THP-1 cells were counted, centrifuged at 1,200rpm (290xg) and the supernatant was removed. Cells were resuspended in buffered DMEM media supplemented with 10% FBS, 1% P/S. Cells were incubated for 2 hours in a humidified environmental chamber (37°C 5% CO_2_). LPS-stimulated cells were then treated with 2.5μg/ml LPS (Invivogen) for up to 6 hours (**Figure S1**). After a total of 8 hours, all cells were collected and total mRNA was extracted using the Omega EZNA Total RNA Kit I, as per the manufacturer’s instructions. RNA samples were diluted in nuclease free water. 10X DNase I Reaction Buffer 1 U/µl DNase I, Amp Grade were added to each tube (Invitrogen, 18068-015). The tubes were incubated for 15 min at 20°C in a thermocycler. 25 mM stock EDTA solution was added to each reaction mixture to inactivate the DNase I, and samples were heated to 65°C for 10 min. Random primer mix (Invitrogen, 48190-011) was added to each tube and the tubes were heated to 70°C for 5 mins, then immediately cooled on ice. M-MLV 5X Reaction Buffer, 10 mM stock dNTP Mix (Bioline, BIO-39044), and M-MLV Reverse Transcriptase (Promega, M170A) were next added. cDNA was synthesised by heating to 37°C for 60 mins. cDNA was stored at -20°C. qRT-PCR was performed using SYBR Green Master Mix (Applied Biosystems) or Taqman Master Mix (Applied Biosystems) and on an ABI 7300 thermocycler (Applied Biosystems). Primer pair sequences used are as follows: β-actin (F: CGACAGGATGCAGAAGGAGA, R: CATCTGCTGGAAGGTGGACA), NR4A1 (F: GTTCTCT GGAGGTCATCCGCAAG, R: GCAGGGACCTTGAGAAG GCCA), NR4A3 (F: CCAAGCCTTAGCCTGCCTGTC, R: AGCCTGTCCCT TACTCTGGTGG). Taqman arrays were used for β-actin and NR4A2 (Applied Biosciences) Relative expression/abundance levels of target gene transcripts were determined using the ΔΔCT method with β-actin as the housekeeping gene. Relative quantification of genes (RQ) is expressed as a fold change over unstimulated control.

### Fluorescent Bead Association Assay

shNT, shNR4A2 and shNR4A3 THP-1 (200,000 cells, in 200µl media per 96 well) were cultured in RPMI media and treated with 1μg/ml LPS for 2hrs followed by addition of 1µm fluorescent beads (Invitrogen, Molecular Probes™, F8819) for a further 2hrs. Plates were then centrifuged at 1,400 rpm for 5 minutes, followed by media removal. The cells were then washed in 200µl PBS, followed by centrifugation and another wash with PBS as described. The cells were then re-suspended in 200µl PBS and analysed for beads association using a fluorescent spectrometer (Molecular Devices, Spectra M2) (Ex535:Em575). A standard curve was generated using known bead quantities and their respective fluorescent value. The number of beads associated with the cells was then extrapolated using the standard curve.

### Cell Migration Assay

shNT, shNR4A2 and shNR4A3 THP-1 cells were cultured in RPMI media and treated with 100ng/ml LPS and untreated controls for 22hrs. Following this, the cells were removed by centrifugation (1,500 rpm for 5 minutes) and conditioned media (600µl) was then removed and incubated +/- anti-MCP1 antibody (2ng/ml) (Biolegend, clone 5D3-F7) for approximately 1hr before being placed in the bottom chamber of a 6.5mm Transwell with 5µm pore polycarbonate membrane insert (Corning^®^ Transwell^®^, CLS3421). 200,000 ‘normal’ THP-1 cells were then placed in the upper chamber in a volume of 100µl media and incubated for 2hrs in a tissue culture incubator. Migrated cells were identified by fixation by placing the inserts in ice-cold pure methanol (MeOH) for 10 minutes, followed by staining using 0.5% crystal violet made up in 25% MeOH. 3 separate fields of view were counted for stained cells at x10 magnification using an inverted microscope and averaged per n number. Microscopic images are representative of each treatment.

### Bioinformatic Analysis

Transcription factor interactome analysis was performed on the Metacore platform (MetaCore+MetaDrug^®^ version 20.3 build 70200) using the list of DEGs obtained by a fold change cut off of 1.4 with a padj < 0.05 (33). Transcription factors were not directly measured in our data but inferred from gene expression signatures based on unbiased predictive analysis of known upstream regulators of DEGs. The transcriptional regulators of DEGs were ranked by *Z*-score cut-off of 2.0 with a padj <0.05.

Selected promoters were examined for the presence of NBREs and NurREs. First, a 5kb region upstream of 8 genes of interest (*ACKRIC2, HLA-F, NCAM1, IFI27, POU4F2, HUNK, CACNA2D1, MCF2*) 1 positive control containing an NBRE (*CCL20*) and 3 negative controls (*TNF, IL-6* and *CCL2*) was extracted using the NCBI genome data and selection viewer. Then, each of these regions were examined for the presence of NBRE and NurRE sequences using a custom NCBI BLAST pairwise comparison (34) and the EMBOSS needle pairwise nucleotide sequence aligner (35). For NBRE detection, only alignments that contained either 100% query coverage, or was missing the first or last arginine in the alignment, was considered indicative of the presence of an NBRE site. For NurRE alignments, the EMBOSS needle scores were reported for each alignment.

### Statistical Analysis

**Figure 5:**
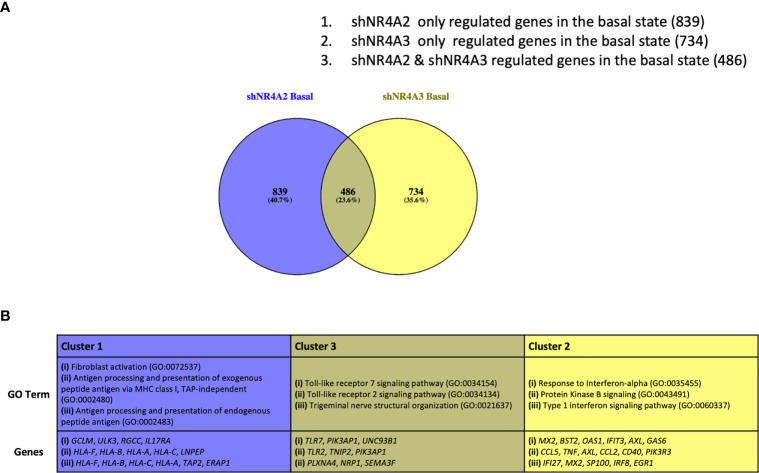
Analysis of gene clusters regulated by *NR4A2* alone, *NR4A3* alone and by both *NR4A2* and *NR4A3*. Venn diagram outlining clusters of genes regulated by *NR4A2* alone (cluster 1), *NR4A2* and *NR4A3* (cluster 2) and *NR4A3* alone (cluster 3) from RNA-seq analysis using a threshold of p-adj </=0.05 for statistical significance **(A)**. Table describing the top 3 most enriched GO terms in genes associated with clusters 1-3 (top panel) in addition to the statistically significantly expressed genes in those clusters associated with the GO terms (bottom panel) **(B)**.

The Wald test was used to generate p-values and log2 fold change values from RNAseq data. For **Figures 1**–**4**, genes with an adjusted p-value < 0.05 and log2 fold change > 1/<-1 were defined as differentially expressed genes. For **Figures 5** and **S2**–**S4**, genes with an adjusted p-value < 0.05 were defined as differentially expressed genes.

**Figure 6:**
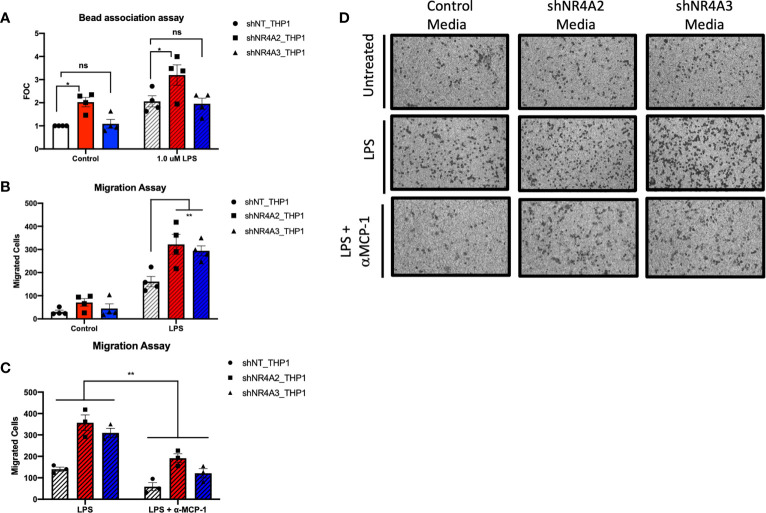
Knockdown of *NR4A2* but not *NR4A3* increases bead association, while knockdown of either *NR4A2* or *NR4A3* increases cell migration. Knockdown of NR4A2 selectively promotes antigen presentation while cell migration is comparably enhanced by loss of NR4A2 or NR4A3. Fluorescent bead association assay in which shNT THP-1 cells, *NR4A2* and *NR4A3* depleted cells were treated +/-1μg/ml LPS for 2hrs followed by addition of 1 μm fluorescent beads source for a further 2hrs. Fluorescence was measured at 535/575 nm. Results expressed as fold over control (FOC) (unstimulated shNT-THP-1) for n= 4 independent experiments **(A)**. Cell migration assay in which shNT-THP-1 cells were exposed for 2hrs to conditioned media from shNT-THP1, *NR4A2* and *NR4A3* depleted cells +/- 100ng/ml LPS (22hrs). Results expressed as migrated cells for n=4 independent experiments **(B)**. Cell migration assay in which shNT-THP-1 cells were exposed for 2hrs to conditioned media from shNT-THP1, *NR4A2* and *NR4A3* depleted cells + 100ng/ml LPS (22hrs). Prior to migration assay conditioned media was treated +/- an anti-MCP1 neutralising antibody (2ng/ml) for approximately 1hr. Results expressed as migrated cells for n=3 independent experiments **(C)**. Note data from non-antibody treated media is also included as part of **(B)** Representative images (10X magnification) of migrated cells +/- LPS, +/- anti-MCP-1 fixed to transwell inserts shown in **(D)**. Statistical analysis (A, B & C) was performed using 2-way ANOVA (assuming sphericity) followed by Sidak’s multiple comparisons test (A&B). **p</= 0.01; ns, p > 0.05.

**Figure 7:**
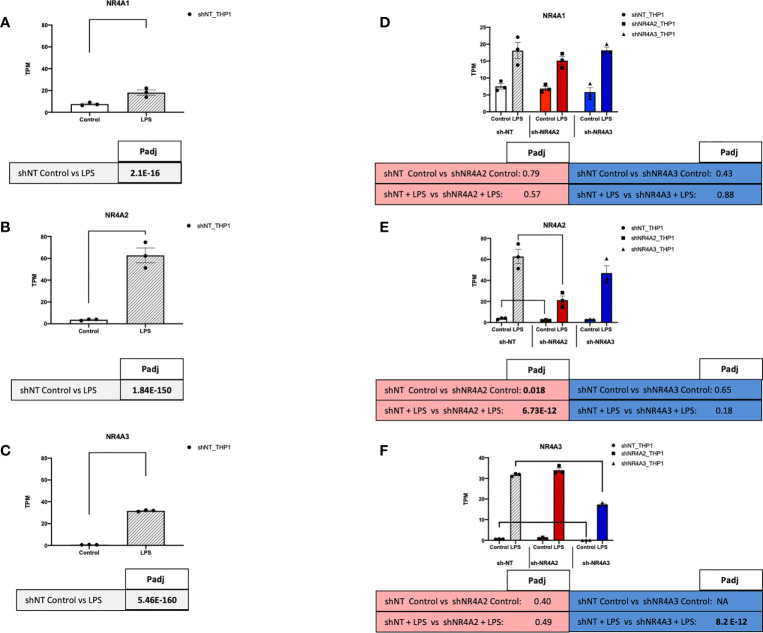
NR4A expression and depletion in THP-1 monocytes. RAW TPM values (mean +/- SEM) extracted from RNA-seq for *NR4A1* **(A)** *NR4A2* **(B)** and *NR4A3* **(C)** in shNT-THP1 cells in the control (unstimulated state) and following LPS treatment (2.5μg/ml for 2hrs) all shown on common axis. RAW TPM values extracted from RNA-seq for *NR4A1* **(D)** *NR4A2* **(E)** and *NR4A3* **(F)** in shNT-THP1 cells, shNR4A2-THP1 cells and shNR4A3-THP1 cells in the control (unstimulated state) and following LPS treatment (2.5μg/ml for 2hrs). N=3 independent experiments. Adjusted p-values (Padj) for specific comparisons are contained in the tables below the TPM data. Statistically significant Padj are denoted in bold font. Comparisons between shNR4A2 cells and control are denoted in the red boxes, comparisons between the shNR4A3 cells and control are denoted in the blue boxes. Statistically significant comparisons are further signposted using bars on the TPM graphs.

Enrichment of gene ontology terms was tested using Fisher exact test [GeneSCF v1.1-p2 for **Figures 1**–**4**, PANTHER Overrepresentation Test (Released 20200728) for **Figure 7** and **Figures S2**–**S4**]. Statistical analysis for **Figure 6** was performed using 2-way ANOVA (assuming sphericity) followed by Sidak’s multiple comparisons test (A&B). Statistical analysis for **Figures S1A–C** was performed using ordinary 1-way ANOVA followed by Dunnett’s multiple comparison test. * denotes p-value </= 0.05, ** denotes p-value of </= 0.01, **** denotes p-value </= 0.0001.

## Results

### *NR4A* Expression and Depletion in THP-1 Monocytes

To assess the function of the nuclear receptors NR4A2 and NR4A3 in human monocytes, we used stable knockdown lines in THP-1 cells. *NR4A1*, *NR4A2* and *NR4A3* RNA expression levels were determined in the presence and absence of LPS. Relatively low Transcripts Per Million (TPM) value (0-10) of each transcript were detected in the basal/unstimulated stated. In the presence of LPS, *NR4A2* and *NR4A3* levels were markedly enhanced, with *NR4A1* being more modestly increased (**Figures 7A–C**). *NR4A1-3* expression increases transiently in response to LPS, peaking after 2-4 hrs of LPS stimulation before dropping at 6hrs (**Figures S1A–D**). Previous work in our laboratory has characterised, albeit not extensively, the shNR4A2 and shNR4A3 cells used within this study (28, 30). These cells display a phenotype congruent with numerous published works using myeloid cells depleted of NR4A2/3 or overexpressing NR4A2/3 (36, 37). In order to compare and contrast the global impact of NR4A2 and NR4A3 on monocytes we used three stable cell lines. shNT-THP1 as our control cell in addition to cells transduced with shNR4A2 and shNR4A3 respectively. The efficacy of the lentiviral knockdown of *NR4A2* and *NR4A3* has been demonstrated using western blot previously (30). Here we validate using RNA-seq quantification. Using raw TPM values, we observed a 66% reduction in *NR4A2* with shNR4A2 in the presence of LPS and a 49% reduction in *NR4A3* with shNR4A3 in the presence of LPS. These reductions were highly statistically significant (Padj 6.73 E-12 and 8.2E-10 for *NR4A2* knockdown and *NR4A3* knockdown respectively. Knockdown of *NR4A2* or *NR4A3* was without an obvious or significant effect on *NR4A1* expression (**Figure 7D**). Previous work from our laboratory has shown a consistent loss of NR4A2 and NR4A3 protein expression in shNR4A2 and shNR4A3 cells in response to LPS compared to shNT (28, 30).

### Knockdown of *NR4A2* Causes Marked Transcriptional Changes

Principal component (PC) analysis exhibited a marked separation between cells transduced with shNT or with shNR4A2, indicating a clear phenotypic distinction of the cell lines with respect to transcriptional profile (**Figure 1A**). This observation was confirmed by differential expression profiles which demonstrated a number of upregulated (243) and downregulated genes (99) using stringent cut-offs (padj<0.05; >2-fold) (**Figure 1B** and **Supplementary Data Table 1**). GO analysis revealed multiple terms associated with the altered transcriptional profile of shNR4A2 cells with ‘GO:0043547 Positive regulation of GTPase activity’, ‘GO:007165 signal transduction’ and ‘GO 0001525 angiogenesis’ being the top 3 most over-represented pathways (**Figure 1C** and **Supplementary Data Table 5**). Novel NR4A2 sensitive genes such as POU Class 4 Homeobox 2 (*POU4F2*) and Hormonally Up-regulated Neu-Associated Kinase (*HUNK*) were the most upregulated and downregulated genes respectively under these conditions with Neural Cell Adhesion Molecule 1 (*NCAM1*), featuring as the 4^th^ most upregulated gene (**Figure 1D**). A heatmap and bi-clustering analysis of *NR4A2* knockdown in THP-1 monocytes is presented in figure (**Figure S2A**).

**Figure 8:**
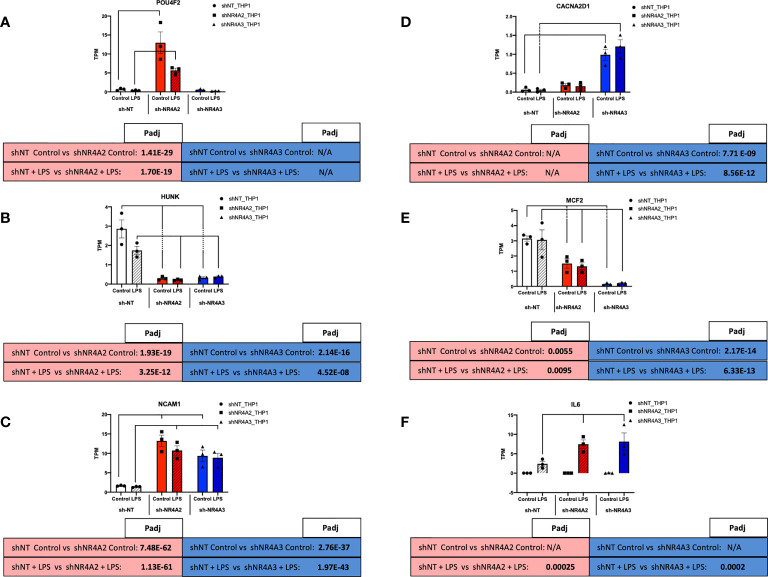
Individual knockdown of *NR4A2* and *NR4A3* alters the expression of distinct and overlapping genes. RAW TPM values (mean +/- SEM) extracted from RNA-seq for *POU4F2* **(A)** *HUNK* **(B)** *NCAM1* **(C)** *CACNA2D1* **(D)** *MCF2* **(E)** and *IL-6* **(F)** in shNT-THP1 cells, shNR4A2-THP1 cells and shNR4A3-THP1 cells in the control (unstimulated state) and following LPS treatment (2.5μg/ml for 2hrs). N=3 independent experiments. Adjusted p-values (Padj) for specific comparisons are contained in the tables below the TPM data. Statistically significant Padj are denoted in bold font. Comparisons between shNR4A2 cells and control are denoted in the red boxes, comparisons between the shNR4A3 cells and control are denoted in the blue boxes. Statistically significant comparisons are further signposted using bars on the TPM graphs.

Similar results were observed when cells transduced with shNR4A2 were stimulated with LPS for 2hrs and compared with shNT cells treated with LPS. PC analysis once more revealed clear separation of the cell types and the co-stimulation with LPS revealed further changes in the transcriptional profile (**Figure 2A**). We observed upregulated genes (351) and downregulated genes (89) due to NR4A2 knockdown (**Figure 2B** and **Supplementary Data Table 2**). GO analysis revealed multiple pathways associated with the altered transcriptional profile of shNR4A2 cells and in the presence of LPS the signal for ‘GO:0001525 angiogenesis’ was markedly enhanced. GO terms associated with positive regulation of GTPase activity and signal transduction remained amongst the top over-represented pathways (**Figure 2C** and **Supplementary Data Table 6**). Several of the most differentially expressed genes were sensitive to shNR4A2 both in the presence and absence of LPS e.g. *POU4F2*, Aldo-Keto Reductase Family 1 Member C2 (*AKR1C2*), *NCAM1* (upregulated) and *HUNK*, Immunoglobulin Superfamily Member 10 (*IGSF10*), Potassium Voltage-Gated Channel Superfamily Q member 5 (*KCNQ5*) (downregulated). The transcription factor associated genes *POU4F2*, and *HUNK* were again the most differentially expressed genes (**Figure 2D**), with *HUNK* not appearing to be particularly sensitive to LPS. Importantly, the cellular response to LPS in all 3 cell lines was extremely robust as evidenced by the marked increase in expression in multiple cytokines and of NR4A family members (**Figures 7**, **8F** and **S10 B, C**).

### Knockdown of *NR4A3* Causes Marked Transcriptional Changes

Principal component (PC) analysis exhibited a marked separation between cells transduced with shNT or with shNR4A3 indicating a clear phenotypic distinction of the cell lines with respect transcriptional profile (**Figure 3A**). This observation was confirmed by differential expression analysis which demonstrated down-regulated genes (149) and upregulated genes (102) due to *NR4A3* knockdown (**Figure 3B** and **Supplementary Data Table 3**). GO analysis revealed multiple pathways associated with the altered transcriptional profile of shNR4A3 cells with a particularly strong signal for ‘GO: 0007155 cell adhesion’ evident. Other enriched GO terms included ‘GO:0006954 inflammatory response’ and ‘GO:0001525 angiogenesis’ (**Figure 3C** and **Supplementary Data Table 7**). This is consistent with the contribution of *NR4A3* deficiency in hematopoietic stem cells to myelopoiesis, monocyte differentiation, and atherosclerosis development *in vivo* (38). Novel NR4A3 sensitive genes such as Calcium Voltage-Gated Channel Auxiliary Subunit Alpha2delta 1 (*CACNA2D1*) and MCD.2 Cell Line Derived Transforming Sequence (*MCF2*) were the most upregulated and downregulated genes respectively under these conditions, with *NCAM1* featuring as the 4^th^ most upregulated gene (**Figure 3D**). A heatmap and bi-clustering analysis of *NR4A3* knockdown in THP-1 monocytes is presented in figure (**Figure S2B**).

The gene expression profile of shNR4A3 treated cells was next tested in the presence of LPS. In contrast to (**Figure 3B**), the presence of LPS in shNR4A3 cells reversed the trend above in favour of upregulated genes (250) compared to downregulated genes (140) (**Figure 4B** and **Supplementary Data Table 4**). ‘GO:0007155 Cell adhesion’ was again the most overrepresented GO term but this enrichment was less pronounced than in the basal state with the two next most enriched GO terms being ‘GO: 0006955 immune response’ and ‘GO:0006954 inflammatory response’ (**Figure 4C** and **Supplementary Data Table 8**). Similar to shNR4A2 above, several of the most differentially expressed genes were sensitive to shNR4A3 both in the presence and absence of LPS e.g. *CACNA2D1*, PR/SET Domain 6 (*PRDM6*), *NCAM1* (upregulated) and *MCF2*, Fidgetin, Microtubule Severing Factor (*FIGN*), Cytoskeleton Associated Protein 4 (*CKAP4*) (downregulated). The calcium channel associated gene *CACNA2D1* was again the most upregulated gene in the shNR4A3 cells in the presence of LPS with Nudix Hydrolase 16 Pseudogene 1 (*NUDT16P1*) being the most downregulated and *FIGN* the second most downregulated (**Figure 4D**).

### Individual Knockdown of *NR4A2* and *NR4A3* Alters the Expression of Distinct and Overlapping Genes

Examining RNA levels (TPM values) from the RNA-seq experiment we observe that *POU4F2* expression is markedly enhanced in response to shNR4A2 in the basal and stimulated state (with LPS suppressing expression somewhat at this time point). Interestingly, shNR4A3 knockdown is without effect on *POU4F2*, suggesting that *POU4F2* is selectively and preferentially regulated by NR4A2 (**Figure 8A**). *HUNK* expression is in contrast significantly suppressed by shNR4A2 and appears to be comparably affected by shNR4A3, suggesting that loss of a single isoform (NR4A2 or A3) is sufficient to affect transcription of this gene (**Figure 8B**). *NCAM1* is statistically significantly increased with both shNR4A2 and shNR4A3 (**Figure 8C**).

Expression of the calcium channel associated gene *CACNA2D1* is very potently enhanced with shNR4A3, with the effects of shNR4A2 being much less so, suggesting that *CACNA2D1* is preferentially regulated by NR4A3 (**Figure 6D**). *MCF2* is very potently suppressed by shNR4A3 in both the basal and stimulated state, while the effects of shNR4A2 on this particular gene are significant but much more modest (**Figure 6E**). *IL-6*, a well characterised NR4A sensitive gene is not detectable in any of the cell lines in the absence of LPS, but its expression is enhanced by both shNR4A2 and shNR4A3 in the stimulated state at this time point (**Figure 6F**). Taken together our genome wide transcriptional analysis of cells depleted of *NR4A2* and *NR4A3* reveals a complex interplay between NR4A2 and NR4A3 with regard to gene expression, such that some genes appear to be preferentially regulated by one NR4A isoform over another, while for other genes loss of either *NR4A2* or *NR4A3* has a very marked effect on transcript levels.

### Analysis of Gene Clusters Regulated by *NR4A2* Alone, *NR4A3* Alone and by Both *NR4A2* and *NR4A3*

Based on our data above we hypothesise that the gene expression profiles observed in **Figures 1**–**4** can be accounted for by distinct roles for NR4A family members in the regulation of specific genes. To test this hypothesis, we re-analysed our dataset to try and define specific clusters of genes. In order to avoid spurious omissions from these lists due to arbitrary fold-change cut-offs, we included any gene that had an adjusted p-value of <0.05 compared to the respective control, regardless of fold change. Using these less stringent filters we identified a larger cohort of genes that were statistically significantly different from their respective controls. These additional genes included in the analysis are shown in volcano plots (**Figure S3A**) (shNR4A2) and (**Figure S3B**) (shNR4A3). **Figure 5A** shows a Venn diagram illustrating the distinct and overlapping genes in unstimulated THP-1 cells from our experiments. In order to test our hypothesis that there are distinct roles for NR4A family members in the regulation of distinct genes we focussed on the following three clusters.

(1) Genes exclusively regulated by shNR4A2 in the basal state (839 genes)
(2) Genes exclusively regulated by shNR4A3 in the basal state (734 genes)
(3) Genes regulated by both shNR4A2 and shNR4A2 in the basal state (486 genes) These genes are listed in (**Supplementary Data Table 11**).

We hypothesised that a closer analysis of these clusters would reveal previously unappreciated individual aspects of NR4A2/NR4A3 biology as well as insight into the pathways in which their roles are interchangeable i.e. loss of one or other of the isoforms affects signalling

In cluster 1 we focused our attention on genes that were solely regulated by shNR4A2 in the basal/unstimulated state. GO analysis of cluster 1 using the Panther database identified a strong enrichment of pathways associated with GO:0072537 ‘fibroblast activation’ and GO:0002480/GO:0002483/GO:0019885, terms associated with “antigen processing and presentation of endogenous peptide antigen”. Most of the genes associated with these GO terms (apart from *ERAP1*) are upregulated by shNR4A2 suggesting activation of fibroblasts and enhanced antigen processing (**Figures S6**–**S8**).

This signature from cluster 1 was distinct from the profile of GO terms identified in cluster 2 which examined those genes exclusively regulated by shNR4A3 in the basal state. In the case of cluster 2, GO analysis identified a strong signature for ‘GO:035455 response to interferon-alpha’ as well as ‘GO:0043491: Protein Kinase B signalling. In general, loss of *NR4A3* resulted in an enhancement of genes associated with the type 1 interferon signalling pathway while there was a mixture of upregulated and downregulated genes in the protein kinase B signalling pathway (**Figures S9**–**S11**).

Notably, those genes associated with cluster 3 have GO terms typically associated with a classical NR4A2/A3 response e.g. TLR dependent signalling GO:0034154/GO:0034134 (**Figures S12**–**S14**).

Taken together these data reveal a distinct divergence of NR4A2/NR4A3 -dependent signalling, with loss of *NR4A2* favouring an increase in antigen presentation and processing while loss of *NR4A3* favours an increase in the interferon response. Interestingly, when a similar analysis is performed on data taken from samples stimulated with LPS, the same dichotomy of signalling is once again observed with respect to antigen presentation (shNR4A2 specifically) and the cellular response to viruses (shNR4A3) (**Figure S4**). Thus, the data strongly points towards the loss of *NR4A2* directing the transcriptional profile of monocytes towards enhanced antigen presentation, and the loss of *NR4A3* shifting the transcriptional profile towards an enhanced interferon/viral response, irrespective of a background stimulus (LPS). Raw TPM values and statistical comparisons for genes associated with Cluster 1, 2 and 3 are shown in the **Supplementary Data** (**S6**–**S14**).

### Transcription Factor Interactome Analysis of Differentially Expressed Transcripts in *NR4A2* and *NR4A3* Depleted Cells

**Figure 9:**
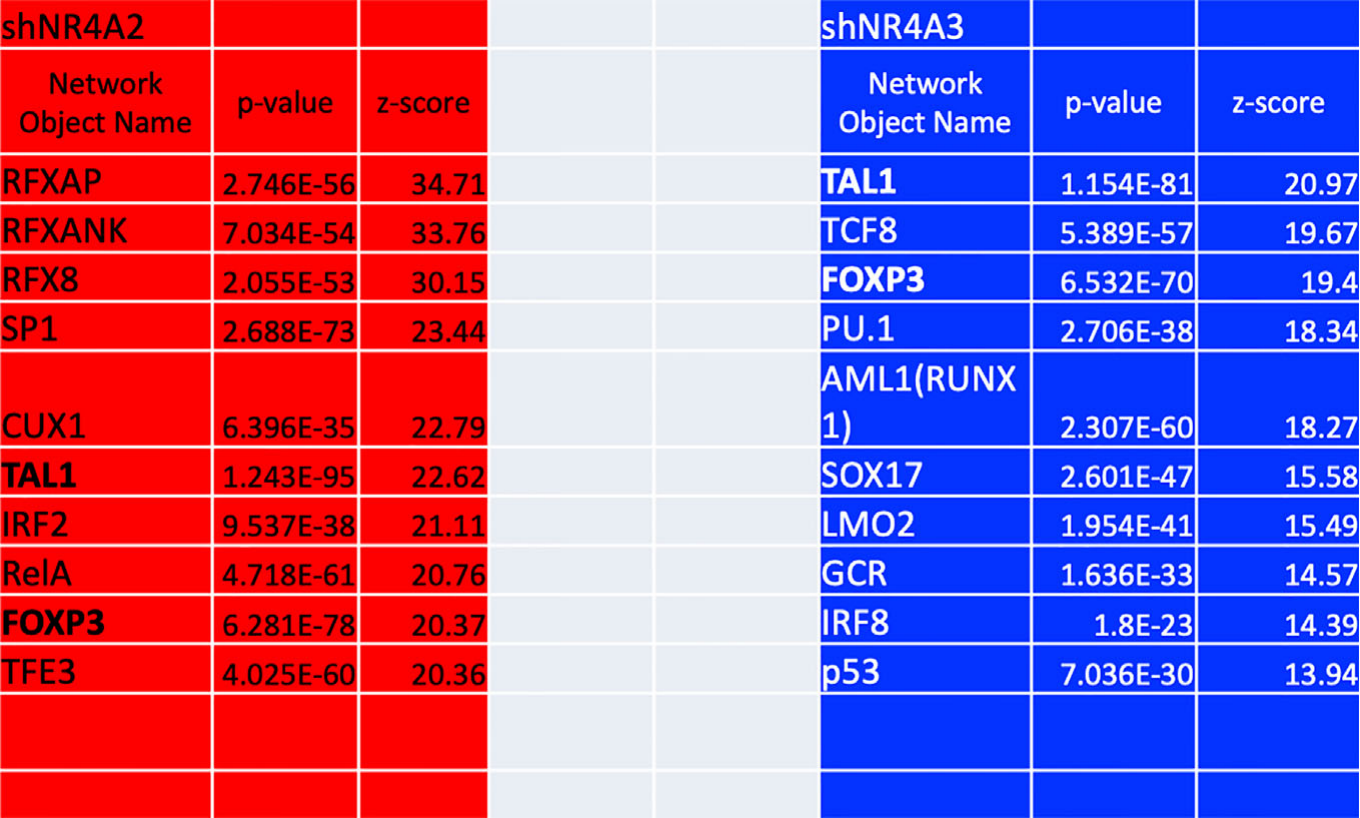
Transcription factor enrichment analysis of genes in *NR4A2* and *NR4A3* depleted cells. Table depicting the top 10 results ranked by z-score from Metacore transcription factor enrichment analysis performed on *NR4A2* (red) and *NR4A3* (blue) depleted cells. Network object name refers to the enriched transcription factor network, p-value refers to the statistical significance of the observation and z-score refers to the distance from the expected mean.

As mentioned above, our gene expression profile data from (**Figures 1**–**5** and **8**) suggests that shNR4A2 and shNR4A3 knockdown elicit distinct (as well as common) roles for NR4A family members in the regulation of specific genes. To infer transcription factors preferentially associated with shNR4A2 or shNR4A3 conditions, we performed Metacore transcription factor interactome analysis using differentially expressed transcripts (padj<0.05; ≥|1.4| fold-change) in shNR4A2 or shNR4A3 expressing cells (**Figure 9**). Interestingly, this analysis revealed a marked divergence amongst the ten most enriched transcription factor pathways when comparing *NR4A2* and *NR4A3* depleted cells. RFX -associated transcription factors (*RFXAP*, *RFXANK* and *RFX8*) ranked amongst the most enriched transcription factors in shNR4A2 cells while they did not feature at all in the top 180 enriched transcription factors in shNR4A3 cells (**Supplementary Data Tables 9**, **10**). In shNR4A3 cells, the most enriched transcription factors were TAL BHLH Transcription Factor 1 Erythroid Differentiation Factor (*TAL1*), Transcription Factor 8 (*TCF8*) and Forkhead box P3 (*FOXP3*). Notably, *TAL1* and *FOXP3* were strongly expressed in the RNAseq data of both shNR4A2 and shNR4A3 cells (**Figure 9**), likely highlighting some of the co-regulated genes in cluster 3. Of the remaining 8 transcription factors enriched in shNR4A3 cells, none were in the top 10 in shNR4A2 cells but several of them were just outside the top ten e.g. *RUNX1* (*AML1*) and *SOX17*. Interestingly, *p53* is in the top 10 for shNR4A3 cells. *NR4A3* has been identified as a novel and direct transcriptional target of p53 which triggers apoptosis and has a tumour- suppressive role (39).

Taken together, this approach implicates several transcription factors that are overrepresented in differential gene expression signatures associated with shNR4A2 and shNR4A3 conditions. The most marked and convincing data is that RFX-associated genes are strikingly associated with shNR4A2 knockdown. Secondly, it is clear that many transcription factors are highly overrepresented in both shNR4A2 and shNR4A3 cells e.g. *TAL1* and *FOXP3*. The transcription factor analysis of shNR4A3 cells does not obviously point to a unique transcriptional regulator of shNR4A3-sensitive genes.

**Figure 10:**
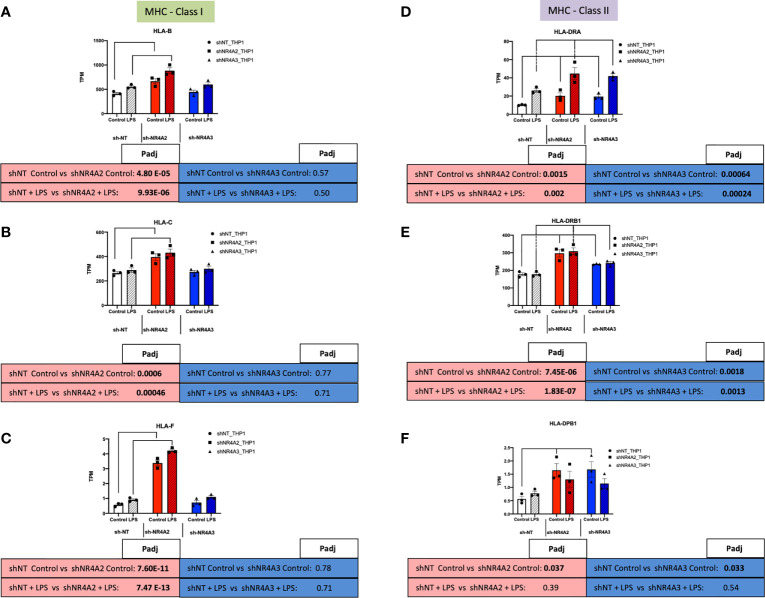
Knockdown of NR4A2 alters MHC Class1 gene expression. RAW TPM values (mean +/- SEM) extracted from RNA-seq for genes associated with MHC-Class I (green rectangles) and MHC-Class II (purple rectangles). Data shown for *HLA-B* **(A)** *HLA-C* **(B)** *HLA-F* **(C)** *HLA-DRA* **(D)** *HLA-DRB1* **(E)** and *HLA-DPB1* **(F)** in shNT-THP1 cells, shNR4A2-THP1 cells and shNR4A3-THP1 cells in the control (unstimulated state) and following LPS treatment (2.5μg/ml for 2hrs). N=3 independent experiments. Adjusted p-values (Padj) for specific comparisons are contained in the tables below the TPM data. Statistically significant Padj are denoted in bold font. Comparisons between shNR4A2 cells and control are denoted in the red boxes, comparisons between the shNR4A3 cells and control are denoted in the blue boxes. Statistically significant comparisons are further signposted using bars on the TPM graphs.

Given the strong signature for RFX-associated genes in our transcription factor enrichment analysis above we hypothesised that downstream genes associated with this transcription factor would map to cluster 1 (shNR4A2 selective) genes (**Figure 5**). Strikingly, this appears to be the case with RFX-associated genes clearly mapping to the HLA-family of genes involved in MHC protein expression and adhesion/presentation immune signalling (**Figure 10**). While HLA-genes associated with both MHC class I and MHC class II were identified as being related to the RFX transcription factors, genes associated with MHC class I were more selectively sensitive to shNR4A2 e.g. *HLA-B*, *HLA-C* and *HLA-F* in particular (**Figures 10A–C**). Thus, our unbiased combined bioinformatic approaches, demonstrated a clear divergence of (i) shNR4A2 cells towards enhanced gene expression of MHC class genes (likely *via* RFX transcription factors) (cluster 1), from (ii) genes that are non-redundantly co-regulated by shNR4A2 and shNR4A3 (cluster 3) (likely *via* transcription factors such as *TAL1*, *FOXP3*), from (iii) genes that are preferentially regulated by shNR4A3 and are associated with an enhanced interferon/viral response.

### Functional Assays That Differentiate Between *shNR4A2* and *shNR4A3*

Our dual bioinformatic approach suggests that transcripts associated with antigen presentation (**Figures 5** and **S4**) and MHC gene expression (**Figure 10**) are preferentially enhanced in shNR4A2 cells. We hypothesised that this increase in genes associated with antigen processing leads to a functional enhancement of antigen engagement. Using a fluorescent bead-based assay, which is a functional assay that models elements of the early stages of antigen presentation, we demonstrate that shNR4A2 cells have a significantly enhanced association with fluorescently tagged beads compared to control cells and shNR4A3 cells (**Figure 6A**).

While some responses/genes appear to be selectively regulated by shNR4A2 or shNR4A3 our RNA-seq data and bioinformatic analysis clearly suggest that some cellular responses are equally/comparably affected by loss of *NR4A2* or *NR4A3*. In a cell migration assay we observe a clear enhancement of cell migration of control THP-1 cells exposed to conditioned media from either shNR4A2 +LPS and shNR4A3 cells +LPS compared to conditioned media from non-target controls +LPS (**Figures 6B, D**). These data suggest that the secretion of chemotactic/pro-migratory factors is enhanced in both *NR4A2* and *NR4A3* depleted cells, and to a similar degree. We next investigated whether the classic chemotactic agent MCP-1 was involved in mediating cell migration in this model. Addition of an anti-MCP-1 neutralising antibody significantly attenuated the migratory phenotype in THP-1 cells suggesting that MCP-1 is at least in part responsible for the enhanced migration observed when cells were grown in conditioned media from shNR4A2 and shNR4A3 cells (**Figures 6C, D**).

## Discussion

NR4A family members are expressed in a range of different tissues and cells and are associated with a host of cellular responses including modulation of inflammation, apoptosis, metabolism and atherogenesis (15). *NR4A1* is most richly expressed in alveolar epithelial cells with expression also evident in blood, innate and adaptive immune cells. *NR4A2* is most richly expressed in granulocytes while *NR4A3* is most richly expressed in cardiomyocytes with lower expression in blood and immune cells (The Human Protein Atlas www.proteinatlas.org). Our data in (**Figures 7A–C**) is in agreement with expression data from wild-type THP-1s reporting that *NR4A1* is expressed the most, followed by *NR4A2* and *NR4A3* respectively in the unstimulated state (The Human Protein Atlas www.proteinatlas.org). As early response genes, *NR4A1*, *NR4A2* and *NR4A3* mRNA expression is enhanced in THP-1s by LPS stimulation at early time points in the region of 2hrs (**Figures 7A–C** and **S1**) consistent with our previous observations in these cells as well as primary PBMCs (30). In addition, all NR4A family members responded to LPS (**Figures 7A–C**) (28, 30, 40). The fold induction in *NR4A2* and *NR4A3* by LPS was much greater than that for *NR4A1* at 2hrs (**Figures 7A–C** and **S1A–C**), which has been reported previously. As mentioned, a direct transcriptomic approach has yet to be utilised to compare and contrast what pathways/targets are regulated by NR4A2 and NR4A3 in both the stimulated and unstimulated state in myeloid cells. Moreover, a comprehensive analysis of what targets specifically affect NR4A3 is particularly underappreciated in immune cells. Here, we sought to use a shRNA approach to determine the transcriptomic effects of NR4A2 & NR4A3 in THP-1 monocyte cells. This knockdown approach was effective in selectively and significantly depleting the RNA levels of *NR4A2* and *NR4A3* with the respective short-hairpin RNA delivered by lentiviral particles (**Figures 7D–F**).

Using an RNA-seq approach we analysed the effect of shNR4A2 and shNR4A3 compared to shNT-THP-1 in the unstimulated state as well as in the presence of LPS. While the transcript levels of *NR4A2* and *NR4A3* are quite low in the unstimulated state, knockdown of these genes resulted in a marked change in baseline transcription (**Figures 1**, **3**). When looking at the effect of *NR4A2* depletion, we observed several novel transcripts differentially expressed in addition to previously described genes e.g. *CTGF* (decreased) (30). GO analysis revealed pathways that have been previously associated with *NR4A2* e.g. signal transduction, angiogenesis, and immune response, but also those that have not been reported to have significant NR4A2 involvement e.g. positive regulation of GTPase activity (**Figure 1C**) [although GTPase activity has been implicated in NR4A1 signalling (41)]. Interestingly, the knockdown of *NR4A3* resulted in a range of differentially expressed genes that were common to those affected by *NR4A2* e.g. *NCAM1*, (**Figure 3C**). GO analysis of *NR4A3* depleted monocytes revealed a particularly striking enrichment for the GO term cell adhesion. Our previous work has identified *CTGF* as a *NR4A3*-senstitive growth factor related to cell adhesion (30). We also must be mindful that NR4As can regulate each other, and this may explain some overlap in terms of downstream pathways within our analysis (30, 42).

LPS is a potent stimulus for monocytes stimulating a host of classic pro-inflammatory markers in our experiment e.g. *TNF* (**Figure S10B**), *CCL2* (**Figure S10C**), *TLR7* (**Figure S12A**) as well as *NR4A2* (**Figure 7B**) and *NR4A3* (**Figure 7C**) themselves. Indeed, *NR4A2* and *NR4A3* have been reported to regulate immune signalling in several ways including acting in concert with NF-kappaB family members (28). To our surprise depletion of *NR4A2* or *NR4A3* in the presence of LPS resulted in a comparatively similar expression profile to the unstimulated. i.e. while LPS clearly altered gene expression in the cells, loss of *NR4A2* or *NR4A3* largely resulted in the same genes and pathways being affected regardless of LPS stimulation. E.g. (i) *POU4F2* and *HUNK* were the most differentially expressed genes in shNR4A2 knockdown cells with and without LPS. (ii) *CACNA2D1* and *MCF2* are the top two most differentially expressed genes in shNR4A3 knockdown cells with and without LPS. (iii) Angiogenesis is in the top 3 enriched GO terms in shNR4A2 knockdown cells with and without LPS (iv) Cell adhesion is the top GO term in shNR4A3 knockdown cells with and without LPS. Taken together these data indicate that NR4As are not only capable of exerting their signalling role against a background of a pro-inflammatory stimulus (LPS) and NF-kappaB activation, but also in the basal/unstimulated state where their depletion has marked transcriptional consequences. These data are consistent with NR4A2 and NR4A3 being constitutively active nuclear receptors and has implications for the understanding of their role in immune cell homeostasis. This observation of distinct and common roles for NR4A2 and NR4A3 is also clearly in evidence in our clustering analysis with remarkable similarity between the clusters identified in (**Figure 5**) (knockdown of *NR4A2* or *NR4A3* in the unstimulated state) and clusters identified in (**Figure S4**) (knockdown of *NR4A2* or *NR4A3* with LPS treatment) E.g. cluster 1 & 4 are both enriched for GO terms associated with antigen processing and presentation and cluster 3 & 6 are both enriched for GO terms associated with cellular response to interferon-alpha. Intriguingly, most studies or reviews published by ourselves and others describe the NR4As as immediate early response genes, activated by a plethora of mediators. This dogma perhaps underestimates their role in the unstimulated state within a given cell type. One could postulate, based on the data presented here that NR4A2 and NR4A3 in the basal state primes the monocytic response to a given stimulus. Interestingly, approximately one quarter of our gene set were only regulated by *NR4A2* or *NR4A3* in the basal/unstimulated state (top left of Venn diagram **Figure S5**). Taken together these data suggest an important and intrinsic role for *NR4A2* and *NR4A3* in monocytes in the unstimulated state.

While NR4A2 and NR4A3 have previously been described to affect the expression of target gene and protein levels to a comparable degree e.g. TNFα and MCP-1 (28), the response of *HUNK* to knockdown of *NR4A2* and *NR4A3* is remarkably similar. Depletion of *NR4A2* or *NR4A3* significantly reduces the levels of *HUNK* in THP-1 monocytes in the presence and absence of LPS. NR4A2 and NR4A3 share significant structural and sequence similarity and regulate genes through direct binding to DNA at specific sites as well as part of a transcriptional regulatory complex involving multiple proteins. Using *HUNK* as an exemplar it is clear that a sub-set of NR4A-sensitive genes are regulated non-redundantly i.e. loss of either *NR4A2* or *NR4A3* is sufficient to attenuate *HUNK* levels. This suggests that NR4A2 and NR4A3 are activators of *HUNK* in the unstimulated state and that NR4A2/NR4A3 heterodimer formation at the promoter of the *HUNK* gene could potentially explain our data. The role of functional redundancy of NR4A family members has been considered previously (43).

In contrast to the pattern observed for *HUNK* with *NR4A2* and *NR4A3* depletion, *POU4F2* gene expression is markedly enhanced with depletion of *NR4A2* alone. This suggests that NR4A2 is normally a repressor of this transcript and that there is de-repression with shNR4A2. Loss of *NR4A3* is completely without effect on *POU4F2*. NR4A2 is known to regulate gene transcription through recruitment of the CoREST repressor complex. This repressor complex can then displace the binding of other transcriptional complexes e.g. NF-kappaB (36). Thus, despite strong sequence similarity a sub-set of genes are selectively regulated by NR4A2. The same is true for *NR4A3* with *CACNA2D1* as an exemplar. Expression of this calcium channel associated gene is markedly enhanced with depletion of *NR4A3* alone. This suggests that NR4A3 is normally a repressor of this transcript and that there is de-repression with shNR4A3. Loss of *NR4A2* is without effect on *CACNA2D1*. NR4A3 is known to regulate gene expression through monomeric binding to NGFI-B Response Elements (NBREs) and *via* homodimers at Nur-responsive elements (NurREs). NR4A3 however, cannot bind the retinoid X receptor (RXR), unlike NR4A1 and NR4A2 which both heterodimerise with RXR to regulate retinoid signalling (20). It is possible that this NR4A3 selectivity is due to the unique NR4A3-interactome required for selective gene expression (recently reviewed by (24)).

To try and better understand the specific patterns of gene regulation with *NR4A2* and *NR4A3* knockdown better we computationally analysed the promoter regions (-5000bp) of key exemplar genes for evidence of NBREs and NurREs. Of the genes we examined (*CACNA2D1*, *HUNK*, *AKR1C2*, *NCAM1*, *HLA-F*, *MCF2*, *IFI27*, *AKR1C2*), *CACNA2D1* was the only gene with an exact (AAAGGTCA) NBRE sequence (-1104bp). *HUNK* (-216bp) and *AKR1C2* (-2949bp) had an NBRE lacking the final nucleotide (AAAGGTC) while *NCAM1* (-1094bp) and *HLA-F* (-3273bp) had an NBRE lacking the first nucleotide (AAGGTCA). The first and last nucleotides of this NBRE sequence are the most dispensable and sequences lacking these nucleotides remain permissive for binding (44). Thus, while several of our genes of interest contain NBRE sequences in their promoter regions, their presence can’t fully explain the pattern of expression we observe. For example, *HUNK* and *AKR1C2* both share a partial NBRE sequence (AAAGGTC), but *HUNK* is clearly regulated by both NR4A2 and NR4A3 (**Figure 8B**) while *AKR1C2* is NR4A2 selective (**Supplementary Data Table 11**). Similarly, *NCAM1* and *HLA-F* both share a different partial NBRE (AAGGTCA) sequence and likewise display differential expression with *NCAM1* regulated by both NR4A2 and NR4A3 (**Figure 8C**) while *HLA-F* is NR4A2 selective (**Figure 10C**). Furthermore, when we screened genes for the NurRE sequence (TGATATTTACCTCCAAAATCCA) we did not observe a perfect alignment in any of our selected 8 genes. Alignment scores similarly for the NurRE furthermore did not differentiate between genes that respond differently to depletion of *NR4A2* and *NR4A3*.

Thus, there is clear evidence of a complex interplay between NR4A2 and NR4A3 with respect to the regulation of gene expression in monocytes. To understand this better we generated the discrete clusters (**Figure 5**) isolating genes that are selectively NR4A2 sensitive (cluster 1), genes that are co-regulated by NR4A2 and NR4A3 (cluster 2) in addition to genes selectively regulated by NR4A3 (cluster 3). This approach clearly segregated genes that were selectively regulated by NR4A2 compared to NR4A3 and in turn identified biological pathways that appear to be preferentially or selectively regulated in each cell type e.g. antigen processing for NR4A2 and response to interferon -alpha for NR4A3. While there is evidence of redundancy with respect to NR4A signalling, Boulet et al. recently reported non-redundant roles for NR4A1 and NR4A3 in murine monocytes (31). This has been linked to differential expression levels of NR4A family members in different cell sub-types but could also be a feature of unique signalling modalities. Thus, our data strongly support the concept of both redundant and non-redundantly regulated NR4A target genes in monocytes.

Taking a complimentary bioinformatic approach, we next used Metacore software to perform transcription factor interactome analysis on significantly differentially expressed transcripts from *NR4A2* and *NR4A3* depleted cells **(Supplementary Data Tables 9**, **10**). We hypothesised that this would provide additional mechanistic insight into how NR4A2-selective signalling differs from NR4A3-selective signalling. Interestingly, Metacore analysis of shNR4A2 cells showed a marked and selective enrichment for RFX associated transcription factors in *NR4A2* depleted cells that was not present in *NR4A3* depleted cells. These data suggest that there is a statistically significant enrichment/over-representation of genes known to be regulated by RFX transcription factors in shNR4A2 cells. RFX transcription factors form a complex that binds the X box motif of certain MHC class II gene promoters leading to transcriptional activation (45). Intriguingly, when we then examined the differentially expressed genes associated with the top GO terms identified in cluster 1 E.g. GO:0002480/GO:0002483/GO:0019885, terms associated with “antigen processing and presentation of endogenous peptide antigen, these same genes were interacted to RFX-transcription factors in our transcription factor analysis. Thus, our two non-biased bioinformatic approaches led to strikingly similar conclusions. Furthermore, our transcription factor analysis suggests that RFX-transcription factors are associated with the activation of MHC associated genes. These RFX-associated genes are shown in (**Figure 10**) and sub-divided into genes related to MHC Class I or MHC Class II signalling. In response to *NR4A2* depletion all of these transcripts are significantly elevated, some with modest fold changes e.g. *HLA-C* (**Figure 10B**) and some more potently e.g. *HLA-F* (**Figure 10C**). These data suggest that NR4A2 is a repressor of MHC gene expression and that loss of *NR4A2* may enhance antigen presentation in *NR4A2* depleted cells.

While our observations regarding RFX-associated genes and *NR4A2* depletion are the most novel findings from the transcription factor interactome analysis there were several other observations of note from these data. Several transcription factors were highly enriched in both *NR4A2* and *NR4A3* depleted cell e.g. *TAL1* and *FOXP3*, consistent with the concept of NR4A2 and NR4A3 having co-regulatory roles for several genes. TAL1 is an activator of cyclin D1 (*CCND1* **)** (46) and VEGFR2 (47) both of which are affected by depletion of *NR4A2* and *NR4A3*. FOXP3 is an inhibitor of *NCAM1* (which is equally affected by depletion of *NR4A2* and *NR4A3* (**Figure 8C** cluster 2) (48, 49) and NR4A family members transactivate the expression of *FOXP3*, which is essential for generation of T regulatory cells (50). Furthermore, another study has identified a role for NR4As and FOXP3 in the regulation of Cytotoxic T- Lymphocyte Associated Protein 4 (*CTLA4*) (51). Thus, there is clearly established cross talk between NR4As and FOXP3. Taken together our transcription factor analysis suggests (i) a selective enrichment of RFX associated genes in cells deficient in *NR4A2* (ii) enrichment of TAL1 and FOXP3 associated genes in both *NR4A2* and *NR4A3* depleted cells and (iii) no clear evidence for a transcription factor selectively/uniquely associated with *NR4A3*.

Our data to date has outlined novel insights into the role of NR4A2 and NR4A3 for gene expression. We next investigated the functional consequences for depletion of these genes in monocytes. Using a fluorometric functional assay that models elements of the early stages of antigen presentation i.e. antigen attachment and/or internalisation we observed a selective increase in fluorescently tagged beads with *NR4A2* depleted cells compared to either control or *NR4A3* depleted cells in the unstimulated state (**Figure 6A**). This selective behaviour of the *NR4A2* depleted cells is aligned with our *in silico* data from (**Figures 5** and **S7**, **S8**) where genes in the *NR4A2* selective cluster 1 were enriched with genes associated with antigen presentation and interacted with RFX transcription factors discussed above (**Figure 9**). Thus, this functional assay supports and helps to validate the bioinformatic analysis of our RNA-seq experiment. Furthermore, our previous study centred on the role of adenosine signalling and the NR4As (28) demonstrated in the exact same cell lines that other elements relating to antigen presentation were selectively enhanced in shNR4A2 cells compared to shNR4A3 cells. The antigen presentation proteins MHC class II (52), CCR7 (53), CD86 and CD80 (54) were all selectively enhanced in shNR4A2 cells compared to shNR4A3 cells in this study (28). We propose that loss of *NR4A2* may change MHC gene expression *via* RFX-dependent transcriptional activity.

Using a distinct cell migration assay, we observed an increase in migrated cells upon exposure to conditioned media from *NR4A2* and *NR4A3* depleted cells treated with LPS (**Figures 6B**, **D**). Thus, both *NR4A2* and *NR4A3* depleted cells are capable of secreting comparable chemotactic agents, (downstream of gene expression changes identified here) that stimulate the migration of monocytes. (**Figures 6C, D**) demonstrates that use of a neutralising antibody targeted to MCP-1 significantly attenuates migration of monocytes in this system. Notably, we have previously reported increased levels of MCP-1 in media taken from shNR4A2 and shNR4A3 cells treated with LPS (30). Interestingly, in our clustering analysis (**Figure S5**), cluster 7 reveals the GO term ‘negative regulation of chemokine-mediated signalling pathway’ as being the most enriched GO term amongst the 276 genes that are NR4A2 and NR4A3 sensitive both in the unstimulated and stimulated state. Thus, our cell migration functional assay further supports and helps to validate our bioinformatic analysis. While, not addressing every pathway implicated in this study, the functional assays in (**Figure 6**) strongly support our bioinformatic approach as a whole.

As mentioned earlier, the NR4As play pivotal roles in numerous inflammatory diseases, and often aberrations in their expression can be both protective or pathogenic depending on the disease in question, as shown in conditions such as atherosclerosis and arthritis (6, 37, 55, 56) Understanding the distinct, and shared processes each NR4A member governs in specific cell types is important when considering this dynamic nature. Building on our informatic data we have functionally confirmed a distinct role for NR4A2 in antigen presentation, a process important in a classically activated monocyte. This role of NR4A2 in regulating such responses has been published in numerous studies (28, 30, 36, 37). Regarding NR4A3 and its proposed distinct role in viral responses, we recall previous work showing NR4A3 is important in dendritic cell responses following exposure to Newcastle disease virus and murine cytomegalovirus (57), and hypothesise such a mechanism may also exist as evidenced here in monocyte cells. Lastly, the ability to modulate the expression of specific NR4As, through exogenous agents, has expanded over the last decade (15, 58). A greater understanding of the specific pathways each NR4A governs, in specific cell types, will better inform how they can be targeted as a therapeutic approach. For example, modulating NR4A2 to alter the inflammatory phenotype of a myeloid cell, or NR4A3 to alter viral responses.

Taken together, these data clearly underscore the ability of our experimental approach to discriminate between signalling selectively regulated by NR4A2 compared to signalling co-regulated by NR4A2 & NR4A3, compared to signalling selectively regulated by NR4A3. This information can be harnessed to better understand the role of the NR4As in disease and inform new therapeutic approaches.

## Limitations

This study has provided significant additional mechanistic insight into the role of NR4A2 and NR4A3 family members in monocytes. In our efforts to try and dissect distinct signalling roles for each NR4A, we segregated data or clustered genes with specific cut-off criteria based on a p-adj of </=0.05. It is possible that with additional experimentation some genes could reach this threshold of significance and consequently move from one cluster to another. Thus, our approach reveals genes that are selective in the context of our study and should not be considered absolute or universally applicable to other settings. We have focused our study on *NR4A2* and *NR4A3* in undifferentiated monocytes. Future work, beyond the scope of the current study could integrate these findings with *NR4A1*, which is known to play an important role in the development of LY6C- ‘patroling monocytes’ (59, 60). NR4A1 levels in monocytes are also known to influence the outcome in models of disease e.g. myocardial infarction (61), neuroinflammation (62) and intestinal disease (63). Indeed, IFI-27 (an anti-viral protein) which was preferentially regulated in shNR4A3 cells (**S11A**) has been demonstrated to be a specific NR4A1 binding protein in vascular cells and serves to downregulate NR4A1-dependent transcriptional activity (64).

THP-1s and monocytes in general express relatively low levels of NR4A family members, with *NR4A3* being the least well expressed of the 3 in the unstimulated state. Our data is most convincing in identifying genes regulated by loss of *NR4A2* in addition to those regulated by loss of both *NR4A2* and *NR4A3*. While we observe a separation of genes associated with loss of NR4A3 alone in the direction of viral responses, we did not observe a unique transcriptional regulator in shNR4A3 cells using Metacore. In general, we observed slightly fewer genes to be regulated by NR4A3 (**Figures 3** and **4**) compared to NR4A2 (**Figures 1**, **2**). This could be explained by a more modest degree of knockdown in our stable cells (49% Vs 66% in the presence of LPS (**Figures 7E, F**) or due to the fact that *NR4A3* is relatively less well expressed in monocytes in the unstimulated state (**Figures 7B, C**).

In a transcriptomic study of this kind, it is not possible to follow-up and validate every pathway or gene that is identified as being of potential interest. Notwithstanding, this study provides an important dataset as a valuable resource for future hypothesis-driven experiments. The data we have generated also has the potential to impact the evolution of NR4A2 and NR4A3 ligands where modulating NR4A agonist/antagonist activity may be appropriate in controlling monocyte differentiation and monocyte specific cell functions. This is a current growth area in the field and it has been nicely demonstrated that several drugs that modulate *NR4A1/NR4A3* expression and function (65).

## Conclusions

In this study we have sought to use incisive bioinformatic analysis of a robust RNA-seq analysis to reveal novel mechanistic insights into the role of NR4A2 and NR4A3 in monocytes in both the unstimulated and stimulated state. These insights are supported by functional studies revealing a divergence in phenotypes between *NR4A2*- and *NR4A3*-depleted cells depending on the specific function e.g. antigen interaction vs cell migration (**Figure 6**). Taken together this study which will enhance our knowledge of the poorly understood pleiotropic NR4A family members *NR4A2* and *NR4A3*.

## Data Availability Statement

The data presented in the study are deposited in the Gene Expression Omnibus (GEO)repository, accession number GSE178391.

## Author Contributions

DP and EC designed the RNA-seq experiments. DP performed the RNA-seq experiments. DP and EC performed the bioinformatic analysis. CM and SA performed experiments relating to the RNA-seq data. MS and JS performed the transcription factor analysis. TH performed the promotor sequence analysis. EM and DC generated the knockdown cell lines and designed the functional assays. DP, EC and DC wrote the manuscript with critical input from all authors. DP and EC edited the final manuscript. All authors contributed to the article and approved the submitted version.

## Funding

EC, DP and CM are funded by an SFI Career Development Award (15/CDA/3490).

MS and JS are funded by HL-147070. SA is funded by the Saudi Arabian Cultural Bureau (SACB).

## Conflict of Interest

The authors declare that the research was conducted in the absence of any commercial or financial relationships that could be construed as a potential conflict of interest.

## Supplementary Material

Supplementary Figure 1 THP-1 cells were stimulated with 2.5μg/ml LPS for 0.5-6hrs. RNA was isolated and qPCR was performed to assess levels of; **(A)** *NR4A1*, **(B)** *NR4A2*, and **(C)** *NR4A3*. *β-actin* was used as a housekeeping gene. Data is presented as mean relative quantification against an unstimulated control (RQ) +/- SD for n=3 individual experiments. Statistical analysis was performed by one-way ANOVA with Dunnett’s multiple comparisons tests. Representative Western blot of NR4A2 and β-actin protein expression in whole cell lysates from THP-1 cells stimulated with 2.5μg/ml LPS for 0-24 hours **(D)**. Image is representative of n=3 individual experiments.

Supplementary Figure 2 Heatmap and biclustering analysis of shNT-THP1 cells compared to *NR4A2* **(A)** and *NR4A3* **(B)** depleted cells. This figure depicts genes that have the top adjusted p-value for statistical significance and were also differentially expressed by a log2fold change of >/=1.

Supplementary Figure 3 Volcano plots for genes that were statistically significantly different (p-adj </=0.05) in *NR4A2* depleted **(A)** and *NR4A3* depleted **(B)** cells compared to shNT-THP1 cells within a log2 fold change range of -1 to +1.

Supplementary Figure 4 Venn diagram outlining clusters of genes regulated by *NR4A2* alone (cluster 4), *NR4A2* and *NR4A3* (cluster 5) and *NR4A3* alone (cluster 6) in the presence of LPS, from RNA-seq analysis using a threshold of p-adj </=0.05 for statistical significance **(A)**. Table describing 3 of the most enriched GO terms in genes associated with clusters 4-6 (top panel) in addition to the statistically significantly expressed genes in those clusters associated with the GO terms (bottom panel) **(B)**.

Supplementary Figure 5 Venn diagram outlining clusters of genes identified when cross-comparing all of our experimental treatments i.e., shNR4A2, shNR4A2 +LPS, shNR4A3, shNR4A3+LPS. Cluster 7 identifies genes that are common to all 4 groups **(A)**. Table describing 4 of the most enriched GO terms in genes associated with cluster 7 (top panel) in addition to the statistically significantly expressed genes in cluster 7 associated with the GO terms (bottom panel) (**B**).

Supplementary Figure 6 RAW TPM values (mean +/- SEM) extracted from RNA-seq for genes associated with the GO term Fibroblast activation genes. Data shown for *GCLM* **(A)** *ULK3* **(B)** *RGCC* **(C)** *IL17RA* in shNT-THP1 cells, shNR4A2-THP1 cells and shNR4A3-THP1 cells in the control (unstimulated state) and following LPS treatment (2.5μg/ml for 2hrs). N=3 independent experiments. In each case *NR4A2* depletion (and not *NR4A3* depletion) in the unstimulated state is statistically significantly different compared to control cells using DESeq2 bioinformatic analysis (cluster 1). Adjusted p-values (Padj) for specific comparisons are contained in the tables below the TPM data. Statistically significant Padj are denoted in bold font. Comparisons between shNR4A2 cells and control are denoted in the red boxes, comparisons between the shNR4A3 cells and control are denoted in the blue boxes. Statistically significant comparisons are further signposted using bars on the TPM graphs.

Supplementary Figure 7 RAW TPM values extracted from RNA-seq for genes associated with the GO term “Antigen RAW TPM values (mean +/- SEM) extracted from RNA-seq for genes associated with the GO term Antigen processing and presentation of exogenous peptide antigen *via* MHC class I, TAP independent. Data shown for *HLA-A* **(A)** *HLA-B* **(B)** *HLA-C* **(C)** *HLA-F* **(D)** *LNPEP* **(E)** in shNT-THP1 cells, shNR4A2-THP1 cells and shNR4A3-THP1 cells in the control (unstimulated state) and following LPS treatment (2.5μg/ml for 2hrs). N=3 independent experiments. In each case *NR4A2* depletion (and not *NR4A3* depletion) in the unstimulated state is statistically significantly different compared to control cells using DESeq2 bioinformatic analysis (cluster 1). Adjusted p-values (Padj) for specific comparisons are contained in the tables below the TPM data. Statistically significant Padj are denoted in bold font. Comparisons between shNR4A2 cells and control are denoted in the red boxes, comparisons between the shNR4A3 cells and control are denoted in the blue boxes. Statistically significant comparisons are further signposted using bars on the TPM graphs.

Supplementary Figure 8 RAW TPM values (mean +/- SEM) extracted from RNA-seq for genes associated with the GO term Antigen processing and presentation of endogenous peptide antigen. Data shown for *HLA-A* **(A)** *HLA-B* **(B)** *HLA-C* **(C)** *HLA-F* **(D)** *TAP2* **(E)** *ERAP1* **(F)** in shNT-THP1 cells, shNR4A2-THP1 cells and shNR4A3-THP1 cells in the control (unstimulated state) and following LPS treatment (2.5μg/ml for 2hrs). N=3 independent experiments. In each case *NR4A2* depletion (and not *NR4A3* depletion) in the unstimulated state is statistically significantly different compared to control cells using DESeq2 bioinformatic analysis (cluster 1). Adjusted p-values (Padj) for specific comparisons are contained in the tables below the TPM data. Statistically significant Padj are denoted in bold font. Comparisons between shNR4A2 cells and control are denoted in the red boxes, comparisons between the shNR4A3 cells and control are denoted in the blue boxes. Statistically significant comparisons are further signposted using bars on the TPM graphs.

Supplementary Figure 9 RAW TPM values (mean +/- SEM) extracted from RNA-seq for genes associated with the GO term response to interferon alpha. Data shown for *MX2* **(A)** *BST2* **(B)** *OAS1* **(C)** *IFIT3* **(D)** *AXL* **(E)** *GAS6* **(F)** in shNT-THP1 cells, shNR4A2-THP1 cells and shNR4A3-THP1 cells in the control (unstimulated state) and following LPS treatment (2.5μg/ml for 2hrs). N=3 independent experiments. In each case *NR4A3* depletion (and not NR4A2 depletion) in the unstimulated state is statistically significantly different compared to control cells using DESeq2 bioinformatic analysis (cluster 2). Adjusted p-values (Padj) for specific comparisons are contained in the tables below the TPM data. Statistically significant Padj are denoted in bold font. Comparisons between shNR4A2 cells and control are denoted in the red boxes, comparisons between the shNR4A3 cells and control are denoted in the blue boxes. Statistically significant comparisons are further signposted using bars on the TPM graphs.

Supplementary Figure 10 RAW TPM values (mean +/- SEM) extracted from RNA-seq for genes associated with the GO term protein kinase B signaling Data shown for *CCL5* **(A)** *TNF* **(B)** *CCL2* **(C)** *CD40* **(D)** *AXL* **(E)** GAS6 **(F)** *PI3KR3* **(G)** *SESN2* **(H)** in shNT-THP1 cells, shNR4A2-THP1 cells and shNR4A3-THP1 cells in the control (unstimulated state) and following LPS treatment (2.5μg/ml for 2hrs). N=3 independent experiments. In each case *NR4A3* depletion (and not *NR4A2* depletion) in the unstimulated state is statistically significantly different compared to control cells using DESeq2 bioinformatic analysis (cluster 2). Adjusted p-values (Padj) for specific comparisons are contained in the tables below the TPM data. Statistically significant Padj are denoted in bold font. Comparisons between shNR4A2 cells and control are denoted in the red boxes, comparisons between the shNR4A3 cells and control are denoted in the blue boxes. Statistically significant comparisons are further signposted using bars on the TPM graphs.

Supplementary Figure 11 RAW TPM values (mean +/- SEM) extracted from RNA-seq for genes associated with the GO term Type 1 interferon signaling pathway. Data shown for *IFI27* **(A)** *MX2* **(B)** *SP100* **(C)** *BST2* **(D)** *MUM1* **(E)** *IRF8* **(F)** *EGR1* **(G)** *SAMHD1* **(H)** *OAS1* **(I)** *IFIT3* **(J)** *ISG15* **(K)** *IRF7* **(L)** *IFIT1* **(M)** *IFI6* **(N)** in shNT-THP1 cells, shNR4A2-THP1 cells and shNR4A3-THP1 cells in the control (unstimulated state) and following LPS treatment (2.5μg/ml for 2hrs). N=3 independent experiments. In each case *NR4A3* depletion (and not *NR4A2* depletion) in the unstimulated state is statistically significantly different compared to control cells using DESeq2 bioinformatic analysis (cluster 2). Adjusted p-values (Padj) for specific comparisons are contained in the tables below the TPM data. Statistically significant Padj are denoted in bold font. Comparisons between shNR4A2 cells and control are denoted in the red boxes, comparisons between the shNR4A3 cells and control are denoted in the blue boxes. Statistically significant comparisons are further signposted using bars on the TPM graphs.

Supplementary Figure 12 RAW TPM values (mean +/- SEM) extracted from RNA-seq for genes associated with the GO term Toll-like receptor 7 signaling pathway. Data shown for *TLR7* **(A)** *PIK3AP1* **(B)** *UNC93B1* **(C)** in shNT-THP1 cells, shNR4A2-THP1 cells and shNR4A3-THP1 cells in the control (unstimulated state) and following LPS treatment (2.5μg/ml for 2hrs). N=3 independent experiments. In each case *NR4A2* depletion and *NR4A3* depletion in the unstimulated state is statistically significantly different compared to control cells using DESeq2 bioinformatic analysis (cluster 3). Adjusted p-values (Padj) for specific comparisons are contained in the tables below the TPM data. Statistically significant Padj are denoted in bold font. Comparisons between shNR4A2 cells and control are denoted in the red boxes, comparisons between the shNR4A3 cells and control are denoted in the blue boxes. Statistically significant comparisons are further signposted using bars on the TPM graphs.

Supplementary Figure 13 RAW TPM values (mean +/- SEM) extracted from RNA-seq for genes associated with the GO term Toll-like receptor 2 signaling pathway. Data shown for *TLR2* **(A)** *TNIP2* **(B)** *PIK3AP1* **(C)** in shNT-THP1 cells, shNR4A2-THP1 cells and shNR4A3-THP1 cells in the control (unstimulated state) and following LPS treatment (2.5μg/ml for 2hrs). N=3 independent experiments. In each case *NR4A2* depletion and *NR4A3* depletion in the unstimulated state is statistically significantly different compared to control cells using DESeq2 bioinformatic analysis (cluster 3). Adjusted p-values (Padj) for specific comparisons are contained in the tables below the TPM data. Statistically significant Padj are denoted in bold font. Comparisons between shNR4A2 cells and control are denoted in the red boxes, comparisons between the shNR4A3 cells and control are denoted in the blue boxes. Statistically significant comparisons are further signposted using bars on the TPM graphs.

Supplementary Figure 14 RAW TPM values (mean +/- SEM) extracted from RNA-seq for genes associated with the GO term Trigeminal Nerve Structure Gene. Data shown for *PLXNA4* **(A)** *NRP1* **(B)** *SEMA3F* **(C)** in shNT-THP1 cells, shNR4A2-THP1 cells and shNR4A3-THP1 cells in the control (unstimulated state) and following LPS treatment (2.5μg/ml for 2hrs). N=3 independent experiments. In each case *NR4A2* depletion and *NR4A3* depletion in the unstimulated state is statistically significantly different compared to control cells using DESeq2 bioinformatic analysis (cluster 3). Adjusted p-values (Padj) for specific comparisons are contained in the tables below the TPM data. Statistically significant Padj are denoted in bold font. Comparisons between shNR4A2 cells and control are denoted in the red boxes, comparisons between the shNR4A3 cells and control are denoted in the blue boxes. Statistically significant comparisons are further signposted using bars on the TPM graphs.

Supplementary Table 1 _shNR4A2 Differential expression analysis.

Supplementary Table 2 _shNR4A2 + LPS Differential expression analysis.

Supplementary Table 3 _shNR4A3 Differential expression analysis.

Supplementary Table 4 _shNR4A3+LPS Differential expression analysis.

Supplementary Table 5 _shNR4A2 Gene ontology analysis.

Supplementary Table 6 _shNR4A2 + LPS Gene ontology analysis.

Supplementary Table 7 _shNR4A3 Gene ontology analysis.

Supplementary Table 8 _shNR4A3+LPS Gene ontology analysis.

Supplementary Table 9 _Metacore analysis table shNR4A2 (full list).

Supplementary Table 10 _Metacore analysis table shNR4A3 (full list).

Supplementary Table 11 _Clusters 1,2,3_Gene lists and Gene ontology analysis.

Supplementary Table 12 _Clusters 4,5,6_Gene lists and Gene ontology analysis.

Supplementary Table 13 _Cluster 7_Gene lists and Gene ontology analysis.

Supplementary Table 14 _THP-1 Gene Background List.
