# Supplementary material for: Transcriptional Profiling of Monocytes Deficient in Nuclear Orphan Receptors NR4A2 and NR4A3 Reveals Distinct Signalling Roles Related to Antigen Presentation and Viral Response
Source: Front Immunol. 2021 Jun 25;12:676644. doi: 10.3389/fimmu.2021.676644 (PMC8267906; doi:10.3389/fimmu.2021.676644)
Supplement: Supplementary Figure 1 — THP-1 cells were stimulated with 2.5μg/ml LPS for 0.5-6hrs. RNA was isolated and qPCR was performed to assess levels of; (A) NR4A1, (B) NR4A2, and (C) NR4A3. β-actin was used as a housekeeping gene. Data is presented as mean relative quantification against an unstimulated control (RQ) +/- SD for n=3 individual experiments. Statistical analysis was performed by one-way ANOVA with Dunnett’s multiple comparisons tests. Representative Western blot of NR4A2 and β-actin protein expression in whole cell lysates from THP-1 cells stimulated with 2.5μg/ml LPS for 0-24 hours (D). Image is representative of n=3 individual experiments. [file Presentation_1.pdf]

Figure. S1

A.

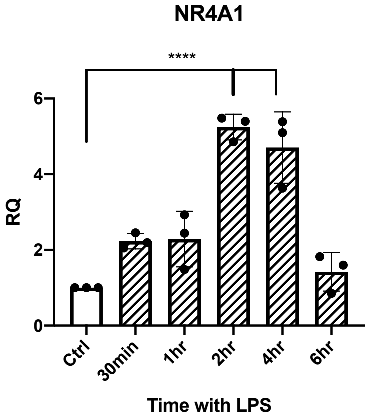

B.

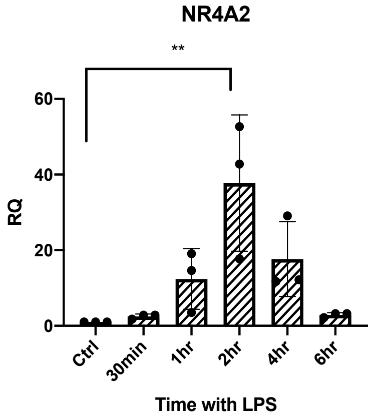

C.

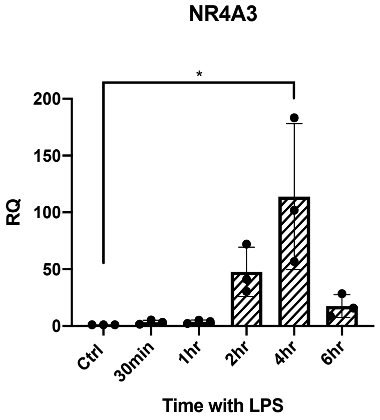

D.

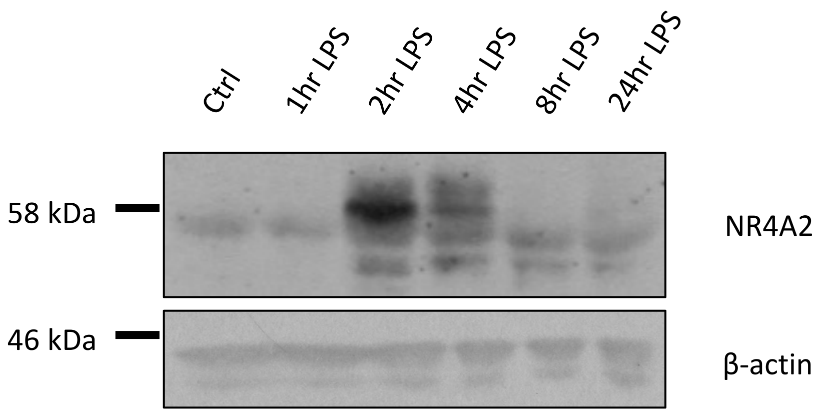

Figure. S2

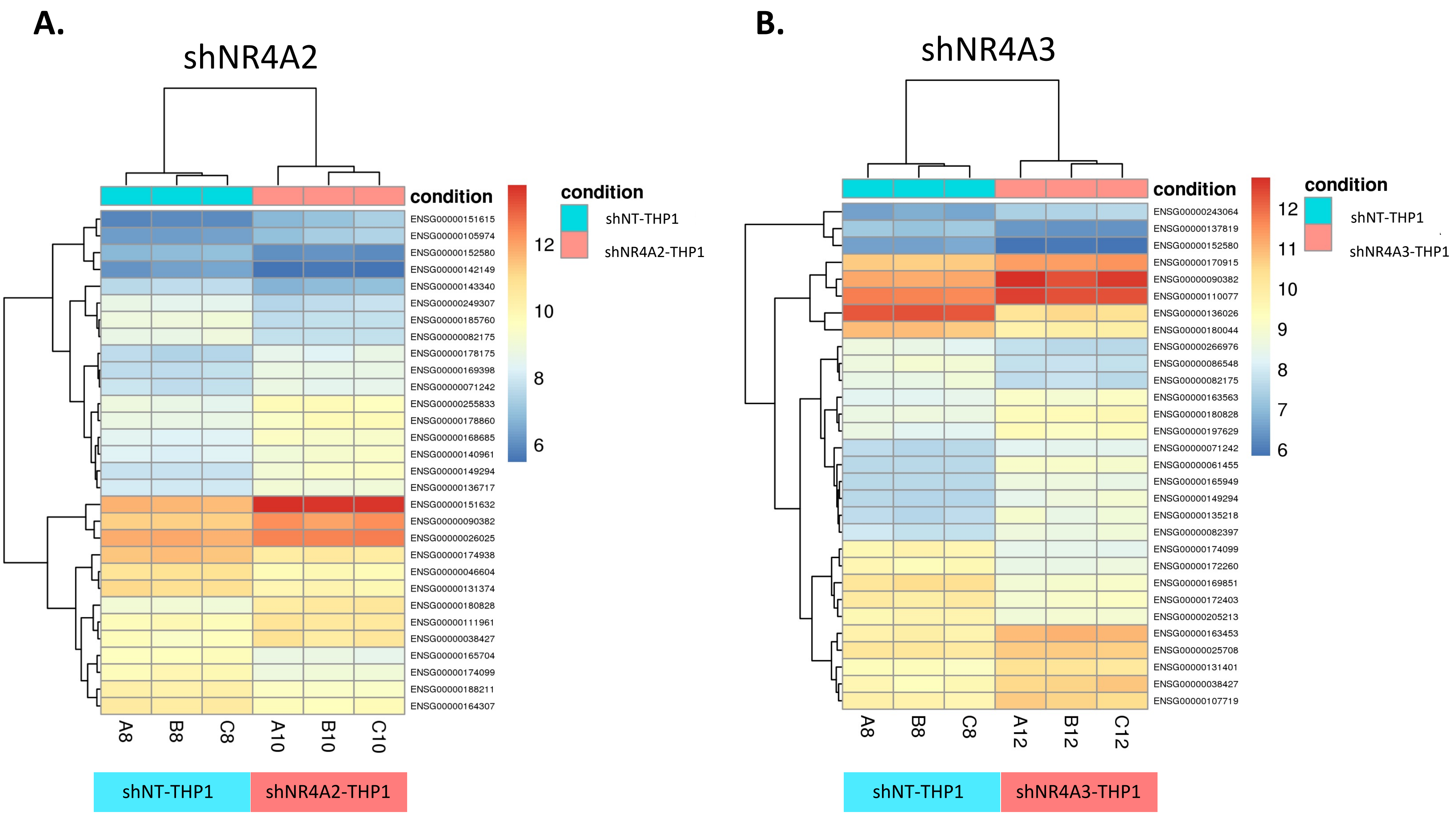
A.
shNR4A2
condition
ENSG00000151615
ENSG00000105974
ENSG00000152580
ENSG00000142149
ENSG00000143340
ENSG00000249307
ENSG00000185760
ENSG00000082175
ENSG00000178175
ENSG00000169398
ENSG00000071242
ENSG00000255833
ENSG00000178860
ENSG00000168685
ENSG00000140961
ENSG00000149294
ENSG00000136717
ENSG00000151632
ENSG00000090382
ENSG00000026025
ENSG00000174938
ENSG00000046604
ENSG00000131374
ENSG00000180828
ENSG00000111961
ENSG00000038427
ENSG00000165704
ENSG00000174099
ENSG00000188211
ENSG00000164307
12
10
8
6
condition
shNT-THP1
shNR4A2-THP1
A8
B8
C8
A10
B10
C10
shNT-THP1
shNR4A2-THP1
B.
shNR4A3
condition
ENSG00000243064
ENSG00000137819
ENSG00000152580
ENSG00000170915
ENSG00000090382
ENSG00000110077
ENSG00000136026
ENSG00000180044
ENSG00000266976
ENSG00000086548
ENSG00000082175
ENSG00000163563
ENSG00000180828
ENSG00000197629
ENSG00000071242
ENSG00000061455
ENSG00000165949
ENSG00000149294
ENSG00000135218
ENSG00000082397
ENSG00000174099
ENSG00000172260
ENSG00000169851
ENSG00000172403
ENSG00000205213
ENSG00000163453
ENSG00000025708
ENSG00000131401
ENSG00000038427
ENSG00000107719
12
11
10
9
8
7
6
condition
shNT-THP1
shNR4A3-THP1
A8
B8
C8
A12
B12
C12
shNT-THP1
shNR4A3-THP1

Figure. S3

A.

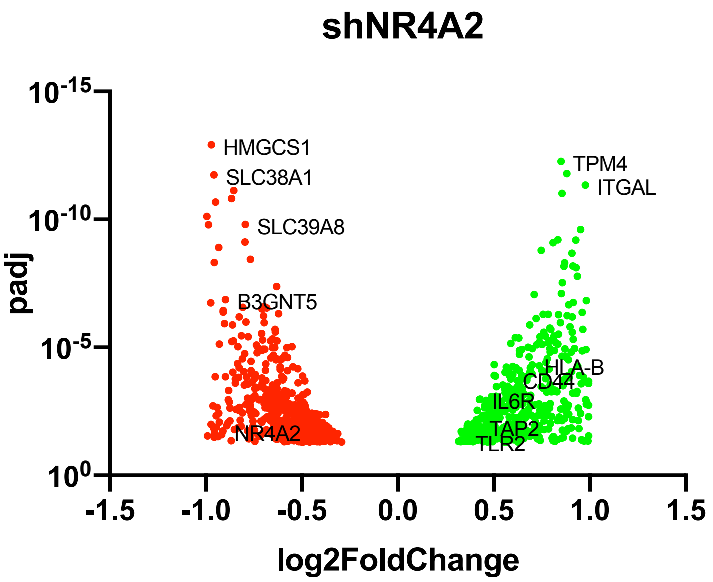
shNR4A2
HMGCS1
SLC38A1
SLC39A8
B3GNT5
NR4A2
TPM4
ITGAL
HLA-B
CD44
IL6R
TAP2
TLR2
padj
log2FoldChange

B.

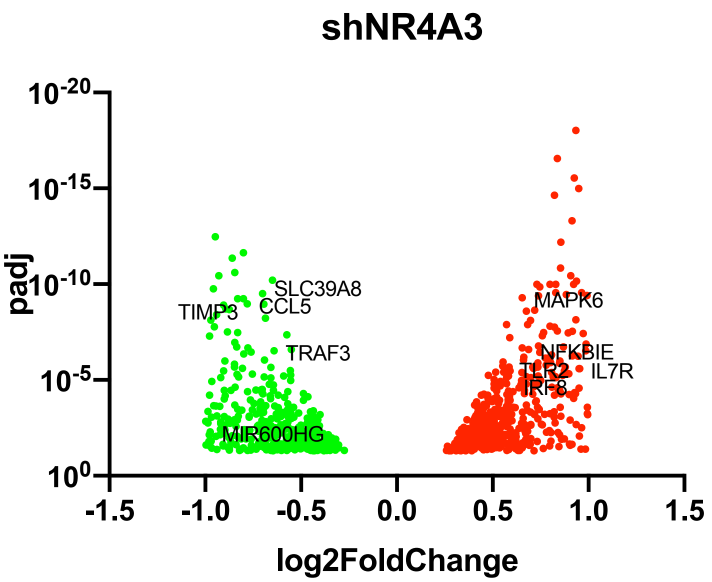
shNR4A3
SLC39A8
TIMP3
CCL5
TRAF3
MIR600HG
MAPK6
NFKBIE
TLR2
IL7R
IRF8
padj
log2FoldChange

Figure. S4

A.

4. shNR4A2 only regulated genes in the presence of LPS (935)
5. shNR4A3 only regulated genes in the presence of LPS (791)
6. shNR4A2 & shNR4A3 regulated genes in presence of LPS (577)

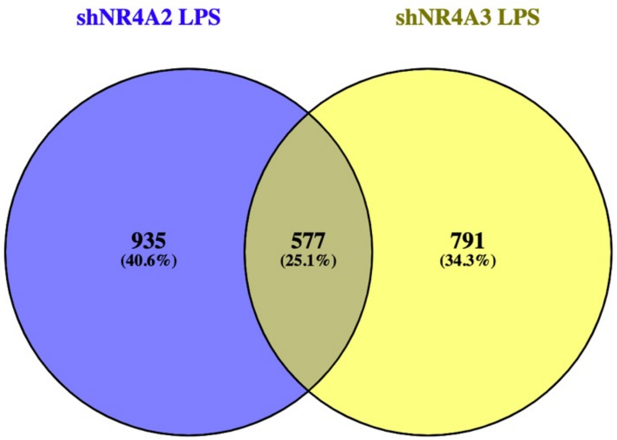

B.

	Cluster 4	Cluster 6	Cluster 5
GO Term	**(i)** Antigen processing and presentation of endogenous peptide antigen via MHC class I via ER pathway, TAP-independent (GO:0002486)<br>**(ii)** Antigen processing and presentation of endogenous peptide antigen via MHC class I via ER pathway (GO:0002484)<br>**(iii)** Antigen processing and presentation of endogenous peptide antigen via MHC class Ib (GO:0002476)	**(i)** T cell activation via T cell receptor contact with antigen bound to MHC molecule on antigen presenting cell (GO:0002291)<br>**(ii)** Regulation of guanyl-nucleotide exchange factor activity (GO:1905097)<br>**(iii)** Regulation of chemokine-mediated signaling pathway (GO:0070099)	**(i)** Positive regulation of monocyte aggregation (GO:1900625)<br>**(ii)** p38MAPK cascade (GO:0038066)<br>**(iii)** Cellular response to interferon-alpha (GO:0035457)
Genes	**(i)** *HLA-C, HLA-E, HLA-B, HLA-F, HLA-A, HLA-G, HLA-H*<br>**(ii)** *HLA-C, HLA-E, HLA-B, HLA-F, HLA-A, HLA-G, HLA-H*<br>**(iii)** *HLA-E, HLA-F, HLA-G, HLA-H, TAP2*	**(i)** *ITGAL, FGL2, ICAM1*<br>**(ii)** *GPSM1, MET, CCPG1, PBXIP1*<br>**(iii)** EDN1, *ROBO1, PADI2, SH2B3*	**(i)** *NR4A3, HAS2, AL133330*<br>**(ii)** *ZFP36, ZFP36L1, LGALS9*<br>**(iii)** *IFIT2, OAS1, IFIT3, AXL, GAS6PHYSIP*

Figure. S5

7. Genes that appear to be equally regulated by shNR4A2 and shNR4A3 in both the basal and stimulated state (276)

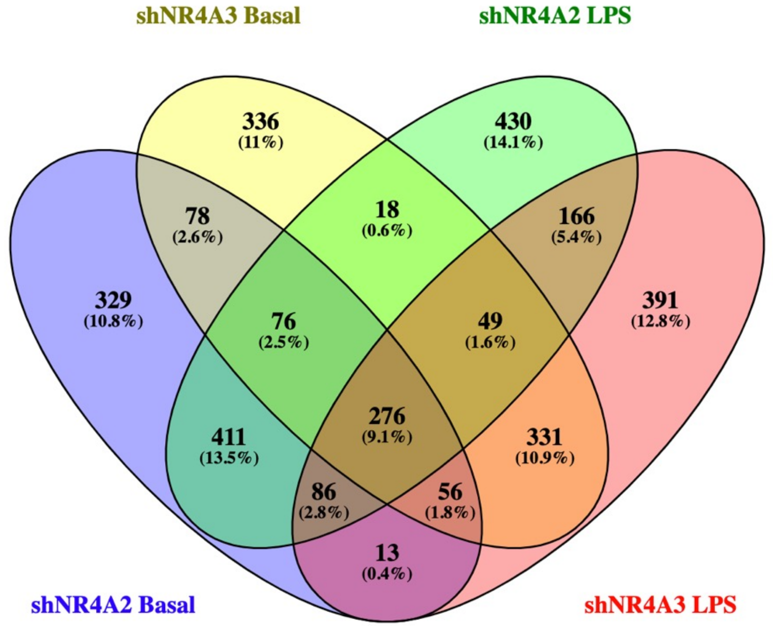

**GO Term**	**(i)** Negative regulation of chemokine-mediated signaling pathway (GO:0070100)<br>**(ii)** Amyloid-beta clearance (GO:0097242)<br>**(iii)** Endothelial cell migration (GO:0043542)<br>**(iv)** I-kappaB kinase/NF-kappaB signaling (GO:0007249)
**Genes**	**(i)** *ROBO1, PADI2, SH2B3*<br>**(ii)** *ITGB2, CD36, ABCA7, IGF1R*<br>**(iii)** *ITGB2, NRP1, DPP4, GPX1, LPXN, NRP1, ROBO1*<br>**(iv)** *TLR7, NLRC3, TIFAB, BCL3, TNIP2, IRAK1BP1*

Figure. S6

Fibroblast activation genes GO:0072537

shNR4A2 selective – Cluster 1

A.

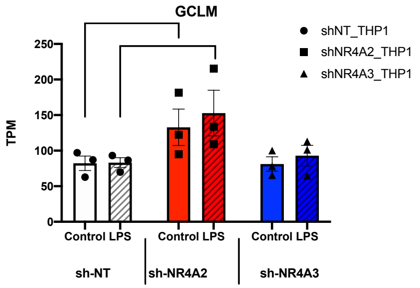

	Padj		Padj
shNT Control vs shNR4A2 Control:	**0.0044**	shNT Control vs shNR4A3 Control:	0.94
shNT + LPS vs shNR4A2 + LPS:	**0.0016**	shNT + LPS vs shNR4A3 + LPS:	0.58

B.

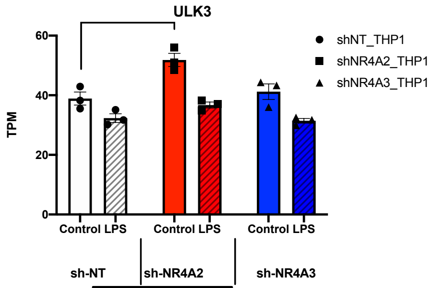

	Padj		Padj
shNT Control vs shNR4A2 Control:	**0.040**	shNT Control vs shNR4A3 Control:	0.71
shNT + LPS vs shNR4A2 + LPS:	0.27	shNT + LPS vs shNR4A3 + LPS:	0.96

C.

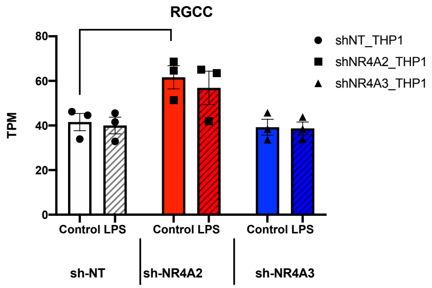

	Padj		Padj
shNT Control vs shNR4A2 Control:	**0.031**	shNT Control vs shNR4A3 Control:	0.95
shNT + LPS vs shNR4A2 + LPS:	0.052	shNT + LPS vs shNR4A3 + LPS:	0.98

D.

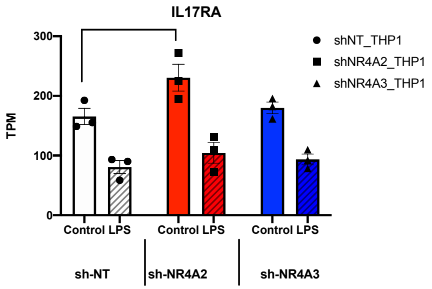

	Padj		Padj
shNT Control vs shNR4A2 Control:	**0.018**	shNT Control vs shNR4A3 Control:	0.49
shNT + LPS vs shNR4A2 + LPS:	0.20	shNT + LPS vs shNR4A3 + LPS:	0.36

Figure. S7

Antigen processing and presentation of exogenous Peptide antigen via MHC class I, TAP independent GO:0002480

shNR4A2 selective – Cluster 1

A.

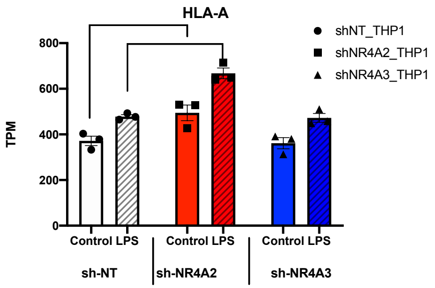

	Padj		Padj
shNT Control vs shNR4A2 Control:	**0.035**	shNT Control vs shNR4A3 Control:	0.99
shNT + LPS vs shNR4A2 + LPS:	**0.00016**	shNT + LPS vs shNR4A3 + LPS:	0.87

B.

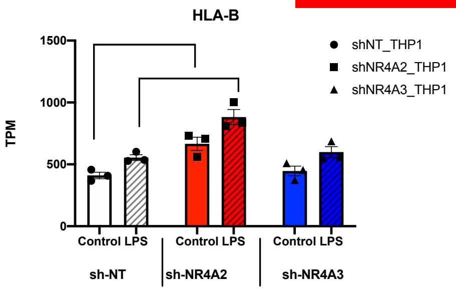

	Padj		Padj
shNT Control vs shNR4A2 Control:	**4.80 E-05**	shNT Control vs shNR4A3 Control:	0.57
shNT + LPS vs shNR4A2 + LPS:	**9.93E-06**	shNT + LPS vs shNR4A3 + LPS:	0.50

C.

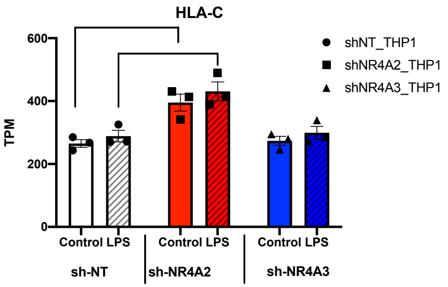

	Padj		Padj
shNT Control vs shNR4A2 Control:	**0.00061**	shNT Control vs shNR4A3 Control:	0.77
shNT + LPS vs shNR4A2 + LPS:	**0.00046**	shNT + LPS vs shNR4A3 + LPS:	0.71

D.

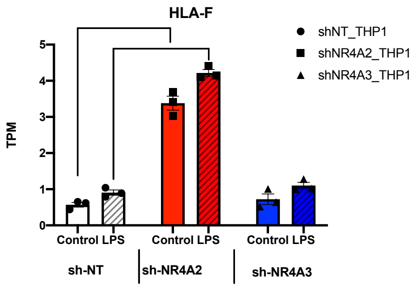

	Padj		Padj
shNT Control vs shNR4A2 Control:	**7.60E-11**	shNT Control vs shNR4A3 Control:	0.78
shNT + LPS vs shNR4A2 + LPS:	**7.47 E-13**	shNT + LPS vs shNR4A3 + LPS:	0.71

E.

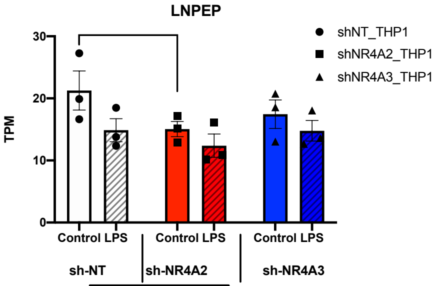

	Padj		Padj
shNT Control vs shNR4A2 Control:	**0.029**	shNT Control vs shNR4A3 Control:	0.47
shNT + LPS vs shNR4A2 + LPS:	0.55	shNT + LPS vs shNR4A3 + LPS:	0.91

Figure. S8

Antigen processing and presentation of endogenous peptide antigen GO:0002483

shNR4A2 selective – Cluster 1

A.

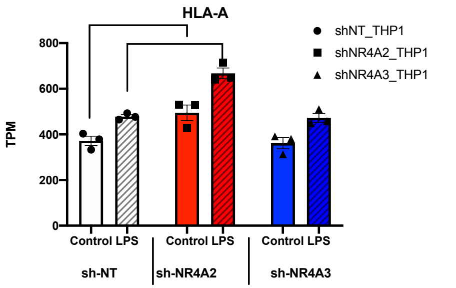

	Padj
shNT Control vs shNR4A2 Control:	**0.035**
shNT + LPS vs shNR4A2 + LPS:	**0.00016**

	Padj
shNT Control vs shNR4A3 Control:	0.99
shNT + LPS vs shNR4A3 + LPS:	0.87

B.

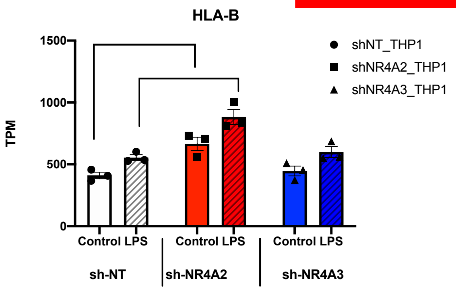

	Padj
shNT Control vs shNR4A2 Control:	**4.80 E-05**
shNT + LPS vs shNR4A2 + LPS:	**9.93E-06**

	Padj
shNT Control vs shNR4A3 Control:	0.57
shNT + LPS vs shNR4A3 + LPS:	0.50

C.

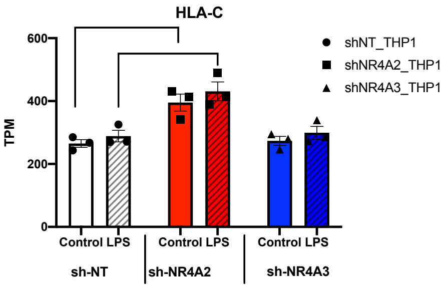

	Padj
shNT Control vs shNR4A2 Control:	**0.00061**
shNT + LPS vs shNR4A2 + LPS:	**0.00046**

	Padj
shNT Control vs shNR4A3 Control:	0.77
shNT + LPS vs shNR4A3 + LPS:	0.71

D.

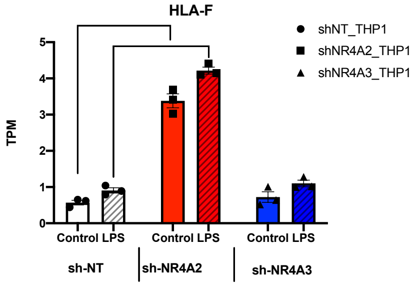

	Padj
shNT Control vs shNR4A2 Control:	**7.60E-11**
shNT + LPS vs shNR4A2 + LPS:	**7.47 E-13**

	Padj
shNT Control vs shNR4A3 Control:	0.78
shNT + LPS vs shNR4A3 + LPS:	0.71

E.

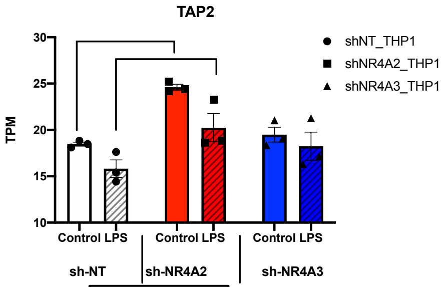

	Padj
shNT Control vs shNR4A2 Control:	**0.012**
shNT + LPS vs shNR4A2 + LPS:	**0.018**

	Padj
shNT Control vs shNR4A3 Control:	0.66
shNT + LPS vs shNR4A3 + LPS:	0.21

F.

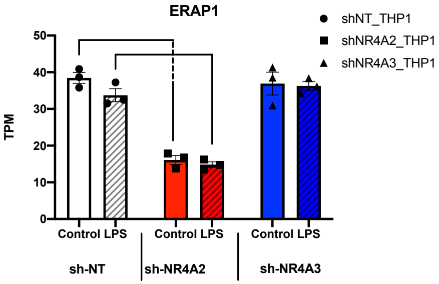

	Padj
shNT Control vs shNR4A2 Control:	**8.34E-21**
shNT + LPS vs shNR4A2 + LPS:	**1.82E-20**

	Padj
shNT Control vs shNR4A3 Control:	0.98
shNT + LPS vs shNR4A3 + LPS:	0.36

Figure. S9

Response to interferon alpha GO:035455

shNR4A3 selective – Cluster 2

A.

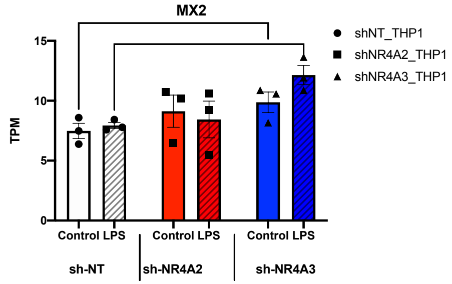

	Padj		Padj
shNT Control vs shNR4A2 Control:	0.39	shNT Control vs shNR4A3 Control:	**0.024**
shNT + LPS vs shNR4A2 + LPS:	0.77	shNT + LPS vs shNR4A3 + LPS	**8.87E-06**

B.

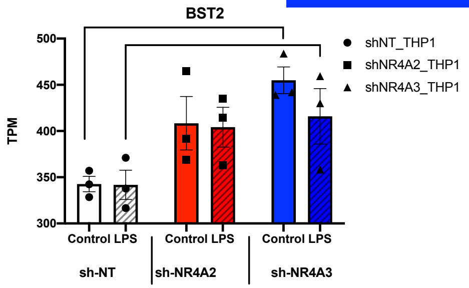

	Padj		Padj
shNT Control vs shNR4A2 Control:	0.25	shNT Control vs shNR4A3 Control:	**0.00019**
shNT + LPS vs shNR4A2 + LPS:	0.095	shNT + LPS vs shNR4A3 + LPS:	**0.045**

C.

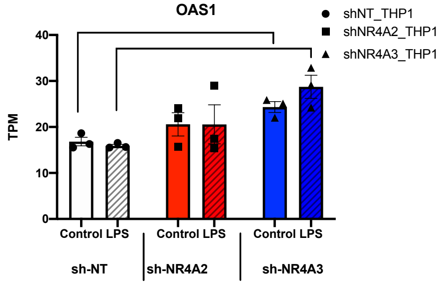

	Padj		Padj
shNT Control vs shNR4A2 Control:	0.31	shNT Control vs shNR4A3 Control:	**0.00011**
shNT + LPS vs shNR4A2 + LPS:	0.24	shNT + LPS vs shNR4A3 + LPS:	**1.64E-07**

D.

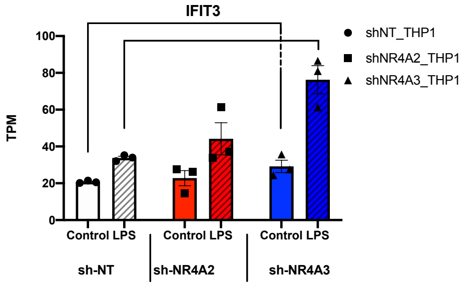

	Padj		Padj
shNT Control vs shNR4A2 Control:	0.80	shNT Control vs shNR4A3 Control:	**0.0083**
shNT + LPS vs shNR4A2 + LPS:	0.20	shNT + LPS vs shNR4A3 + LPS:	**6.84E-12**

E.

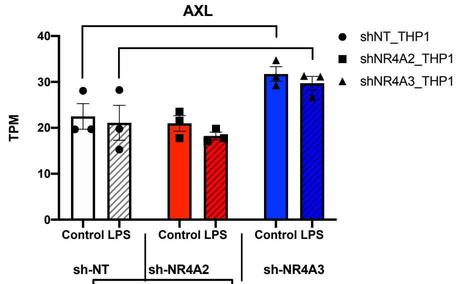

	Padj		Padj
shNT Control vs shNR4A2 Control:	0.829	shNT Control vs shNR4A3 Control:	**0.0076**
shNT + LPS vs shNR4A2 + LPS:	0.747	shNT + LPS vs shNR4A3 + LPS:	**0.039**

F.

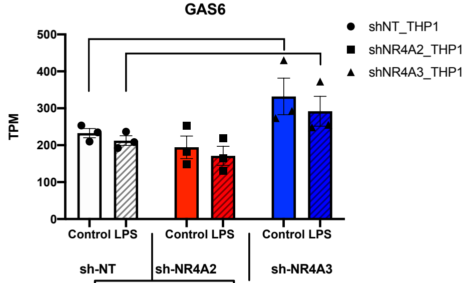

	Padj		Padj
shNT Control vs shNR4A2 Control:	0.439	shNT Control vs shNR4A3 Control:	**0.0068**
shNT + LPS vs shNR4A2 + LPS:	0.494	shNT + LPS vs shNR4A3 + LPS:	**0.026**

Figure. S10

Protein Kinase B Signaling GO:0043491

shNR4A3 selective – Cluster 2

A.

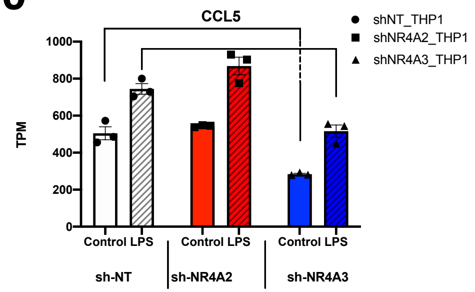

	Padj
shNT Control vs shNR4A2 Control:	0.72
shNT + LPS vs shNR4A2 + LPS:	0.20

	Padj
shNT Control vs shNR4A3 Control:	**1.06E-09**
shNT + LPS vs shNR4A3 + LPS:	**0.0101**

B.

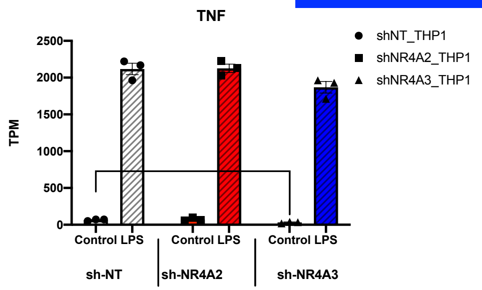

	Padj
shNT Control vs shNR4A2 Control:	0.78
shNT + LPS vs shNR4A2 + LPS:	0.81

	Padj
shNT Control vs shNR4A3 Control:	**3.45E-05**
shNT + LPS vs shNR4A3 + LPS:	0.65

C.

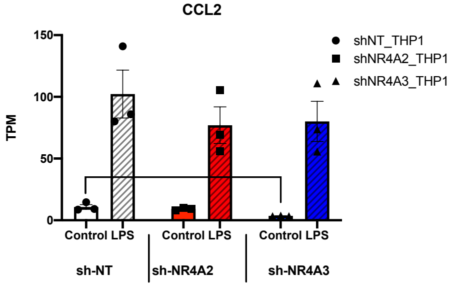

	Padj
shNT Control vs shNR4A2 Control:	0.67
shNT + LPS vs shNR4A2 + LPS:	0.51

	Padj
shNT Control vs shNR4A3 Control:	**4.07E-06**
shNT + LPS vs shNR4A3 + LPS:	0.79

D.

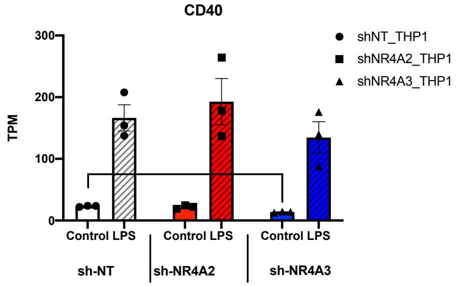

	Padj
shNT Control vs shNR4A2 Control:	0.87
shNT + LPS vs shNR4A2 + LPS:	0.58

	Padj
shNT Control vs shNR4A3 Control:	**0.00014**
shNT + LPS vs shNR4A3 + LPS:	0.79

E.

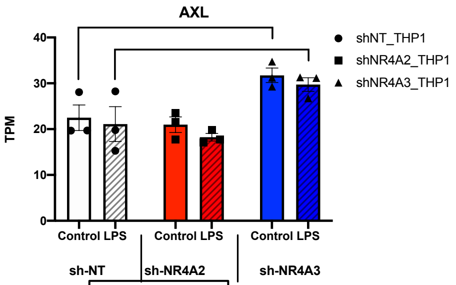

	Padj
shNT Control vs shNR4A2 Control:	0.83
shNT + LPS vs shNR4A2 + LPS:	0.75

	Padj
shNT Control vs shNR4A3 Control:	**0.0076**
shNT + LPS vs shNR4A3 + LPS:	**0.039**

F.

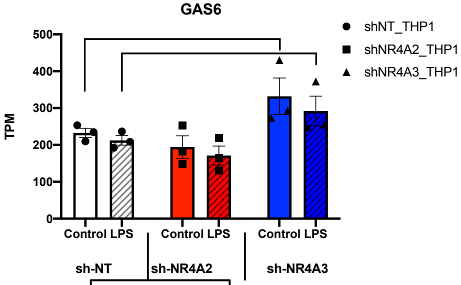

	Padj
shNT Control vs shNR4A2 Control:	0.44
shNT + LPS vs shNR4A2 + LPS:	0.49

	Padj
shNT Control vs shNR4A3 Control:	**0.0068**
shNT + LPS vs shNR4A3 + LPS:	**0.026**

Figure. S10

Protein Kinase B Signaling GO:0043491

G.

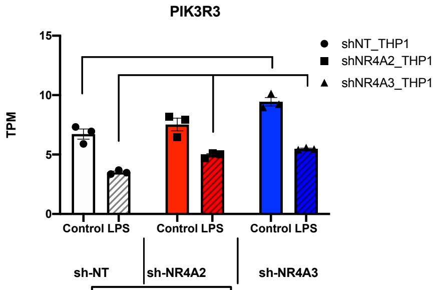

	Padj		Padj
shNT Control vs shNR4A2 Control:	0.65	shNT Control vs shNR4A3 Control:	**0.0035**
shNT + LPS vs shNR4A2 + LPS:	**0.018**	shNT + LPS vs shNR4A3 + LPS:	**0.00068**

H.

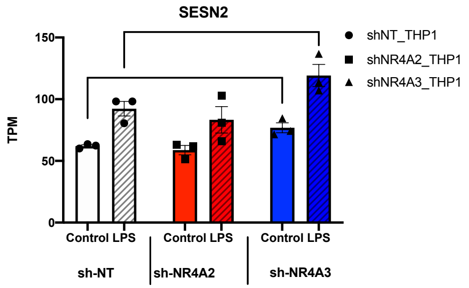

	Padj		Padj
shNT Control vs shNR4A2 Control:	0.85	shNT Control vs shNR4A3 Control:	**0.018**
shNT + LPS vs shNR4A2 + LPS:	0.86	shNT + LPS vs shNR4A3 + LPS:	**0.025**

Figure. S11

Type 1 interferon signaling pathway GO:0060337

shNR4A3 selective – Cluster 2

A.

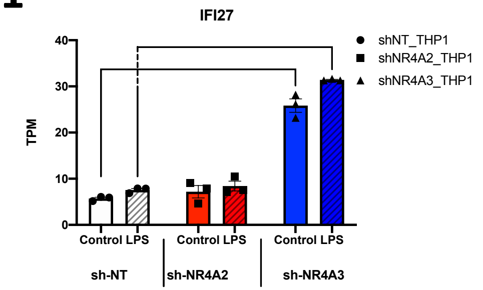

	Padj		Padj
shNT Control vs shNR4A2 Control:	0.48	shNT Control vs shNR4A3 Control:	**1.47E-45**
shNT + LPS vs shNR4A2 + LPS:	0.63	shNT + LPS vs shNR4A3 + LPS:	**2.2E-48**

B.

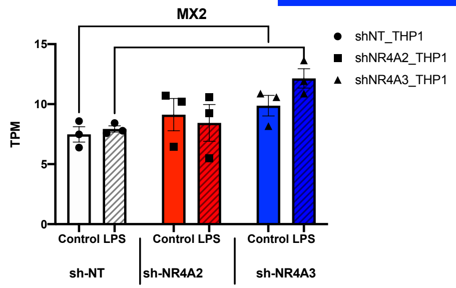

	Padj		Padj
shNT Control vs shNR4A2 Control:	0.39	shNT Control vs shNR4A3 Control:	**0.024**
shNT + LPS vs shNR4A2 + LPS:	0.77	shNT + LPS vs shNR4A3 + LPS	**8.87E-06**

C.

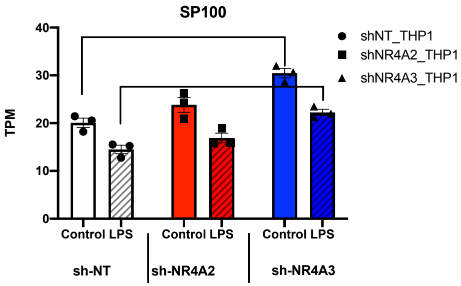

	Padj		Padj
shNT Control vs shNR4A2 Control:	0.24	shNT Control vs shNR4A3 Control:	**2.08E-07**
shNT + LPS vs shNR4A2 + LPS:	0.13	shNT + LPS vs shNR4A3 + LPS:	**5.35E-08**

D.

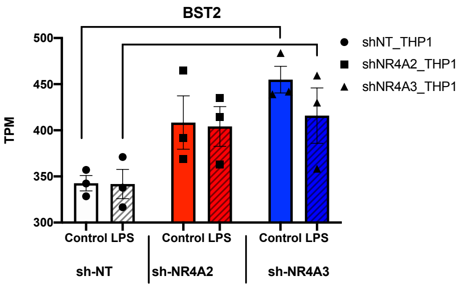

	Padj		Padj
shNT Control vs shNR4A2 Control:	0.25	shNT Control vs shNR4A3 Control:	**0.00019**
shNT + LPS vs shNR4A2 + LPS:	0.095	shNT + LPS vs shNR4A3 + LPS:	**0.045**

E.

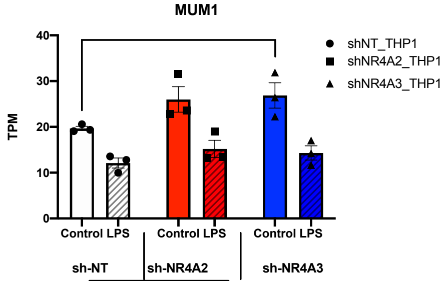

	Padj		Padj
shNT Control vs shNR4A2 Control:	0.067	shNT Control vs shNR4A3 Control:	**0.0021**
shNT + LPS vs shNR4A2 + LPS:	0.12	shNT + LPS vs shNR4A3 + LPS:	0.24

F.

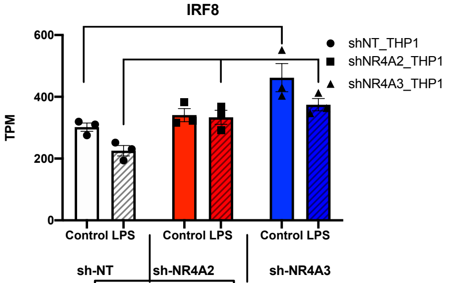

	Padj		Padj
shNT Control vs shNR4A2 Control:	0.51	shNT Control vs shNR4A3 Control:	**4.08E-06**
shNT + LPS vs shNR4A2 + LPS:	**1.31E-5**	shNT + LPS vs shNR4A3 + LPS:	**2.81E-11**

Figure. S11

Type 1 interferon signaling pathway GO:0060337

shNR4A3 selective – Cluster 2

G.

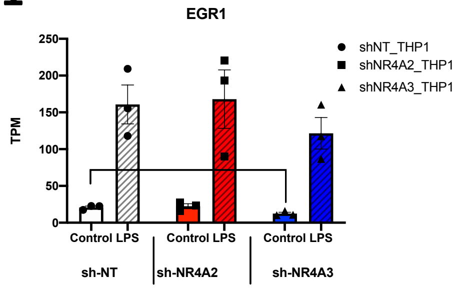

	Padj
shNT Control vs shNR4A2 Control:	0.85
shNT + LPS vs shNR4A2 + LPS:	0.92

	Padj
shNT Control vs shNR4A3 Control:	**0.0030**
shNT + LPS vs shNR4A3 + LPS:	0.35

H.

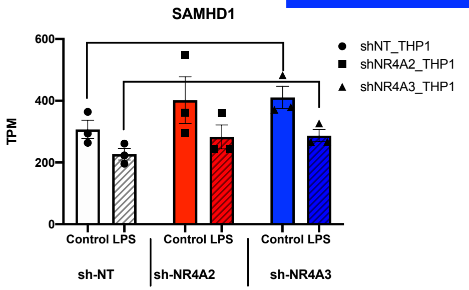

	Padj
shNT Control vs shNR4A2 Control:	0.20
shNT + LPS vs shNR4A2 + LPS:	0.20

	Padj
shNT Control vs shNR4A3 Control:	**0.012**
shNT + LPS vs shNR4A3 + LPS:	**0.048**

I.

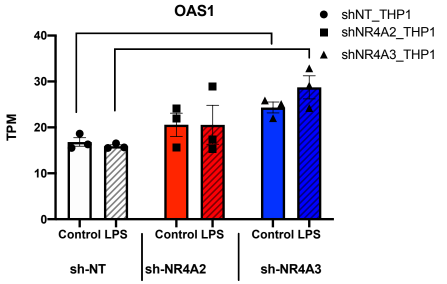

	Padj
shNT Control vs shNR4A2 Control:	0.31
shNT + LPS vs shNR4A2 + LPS:	0.24

	Padj
shNT Control vs shNR4A3 Control:	**0.00011**
shNT + LPS vs shNR4A3 + LPS:	**1.64E-07**

J.

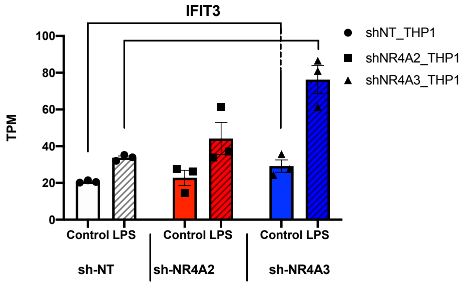

	Padj
shNT Control vs shNR4A2 Control:	0.80
shNT + LPS vs shNR4A2 + LPS:	0.20

	Padj
shNT Control vs shNR4A3 Control:	**0.0083**
shNT + LPS vs shNR4A3 + LPS:	**6.84E-12**

K.

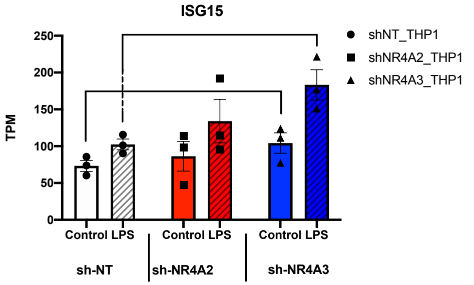

	Padj
shNT Control vs shNR4A2 Control:	0.82
shNT + LPS vs shNR4A2 + LPS:	0.25

	Padj
shNT Control vs shNR4A3 Control:	**0.016**
shNT + LPS vs shNR4A3 + LPS:	**7.41E-06**

L.

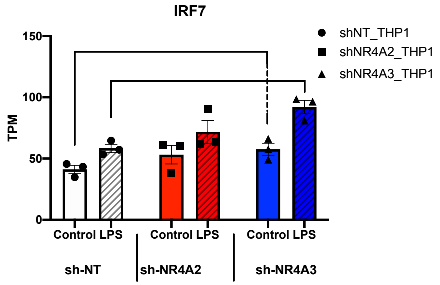

	Padj
shNT Control vs shNR4A2 Control:	0.23
shNT + LPS vs shNR4A2 + LPS:	0.24

	Padj
shNT Control vs shNR4A3 Control:	**0.0042**
shNT + LPS vs shNR4A3 + LPS:	**3.4E-05**

Figure. S11

Type 1 interferon signaling pathway GO:0060337

shNR4A3 selective – Cluster 2

M.

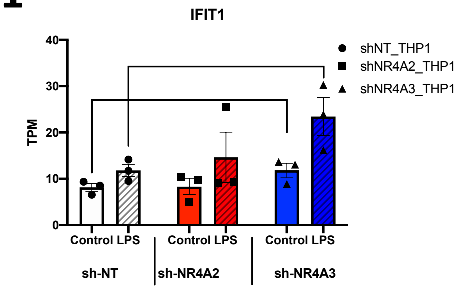

	Padj		Padj
shNT Control vs shNR4A2 Control:	0.97	shNT Control vs shNR4A3 Control:	**0.025**
shNT + LPS vs shNR4A2 + LPS:	0.77	shNT + LPS vs shNR4A3 + LPS:	**0.00011**

N.

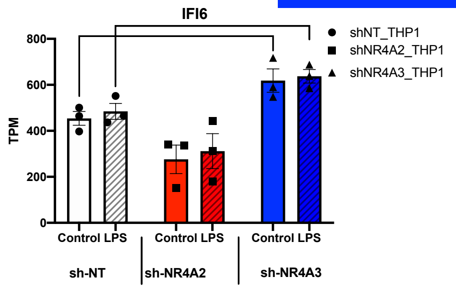

	Padj		Padj
shNT Control vs shNR4A2 Control:	0.26	shNT Control vs shNR4A3 Control:	**0.0086**
shNT + LPS vs shNR4A2 + LPS:	0.12	shNT + LPS vs shNR4A3 + LPS:	**0.013**

Figure. S12

Toll-like receptor 7 signaling pathway GO:0034154

shNR4A2/A3 sensitive – Cluster 3

A.

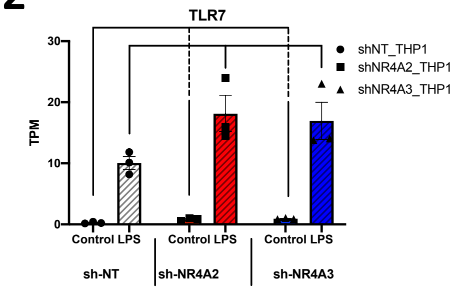

	Padj
shNT Control vs shNR4A2 Control:	**0.042**
shNT + LPS vs shNR4A2 + LPS:	**0.0011**

	Padj
shNT Control vs shNR4A3 Control:	**0.013**
shNT + LPS vs shNR4A3 + LPS:	**0.0034**

B.

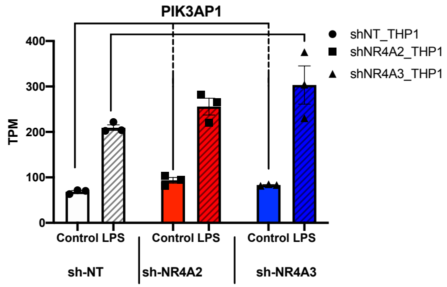

	Padj
shNT Control vs shNR4A2 Control:	**0.0047**
shNT + LPS vs shNR4A2 + LPS:	0.12

	Padj
shNT Control vs shNR4A3 Control:	**0.011**
shNT + LPS vs shNR4A3 + LPS:	**0.0094**

C.

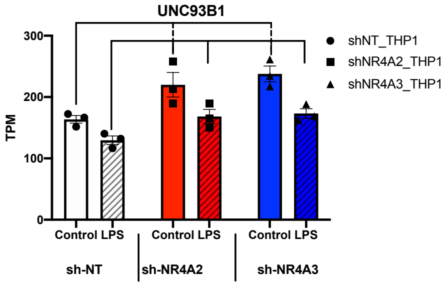

	Padj
shNT Control vs shNR4A2 Control:	**0.035**
shNT + LPS vs shNR4A2 + LPS:	**0.0041**

	Padj
shNT Control vs shNR4A3 Control:	**6.20E-06**
shNT + LPS vs shNR4A3 + LPS:	**0.00012**

Figure. S13

Toll-like receptor 2 signaling pathway GO:0034134

shNR4A2/A3 sensitive – Cluster 3

A.

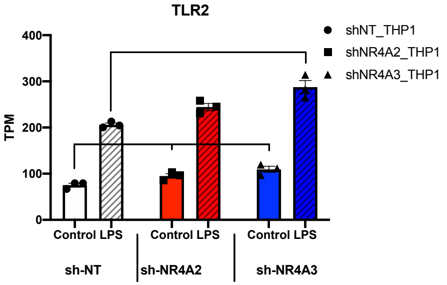

	Padj		Padj
shNT Control vs shNR4A2 Control:	**0.046**	shNT Control vs shNR4A3 Control:	**3.33E-06**
shNT + LPS vs shNR4A2 + LPS:	0.079	shNT + LPS vs shNR4A3 + LPS:	**8.74E-05**

B.

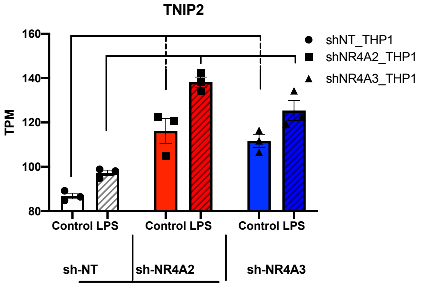

	Padj		Padj
shNT Control vs shNR4A2 Control:	**0.0123**	shNT Control vs shNR4A3 Control:	**0.0029**
shNT + LPS vs shNR4A2 + LPS:	**5.63E-05**	shNT + LPS vs shNR4A3 + LPS:	**0.0020**

C.

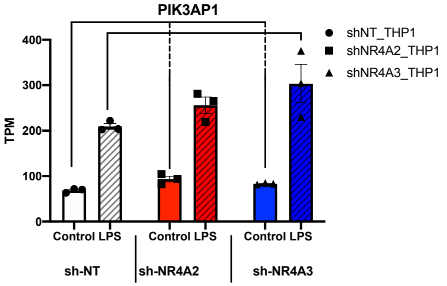

	Padj		Padj
shNT Control vs shNR4A2 Control:	**0.0047**	shNT Control vs shNR4A3 Control:	**0.011**
shNT + LPS vs shNR4A2 + LPS:	0.12	shNT + LPS vs shNR4A3 + LPS:	**0.0094**

Figure. S14

Trigeminal Nerve Structure Gene GO:0021637

shNR4A2/A3 sensitive – Cluster 3

A.

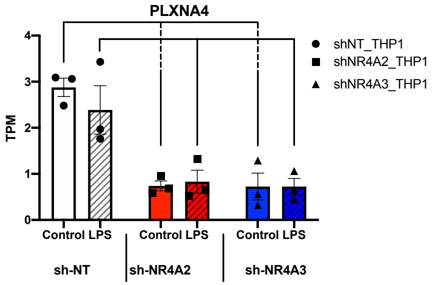

	Padj
shNT Control vs shNR4A2 Control:	**1.39E-16**
shNT + LPS vs shNR4A2 + LPS:	**0.00015**

	Padj
shNT Control vs shNR4A3 Control:	**0.0054**
shNT + LPS vs shNR4A3 + LPS:	**6.77E-07**

B.

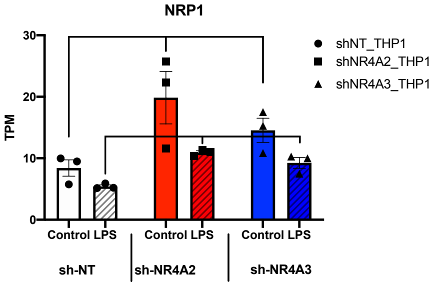

	Padj
shNT Control vs shNR4A2 Control:	**0.022**
shNT + LPS vs shNR4A2 + LPS:	**2.4E-12**

	Padj
shNT Control vs shNR4A3 Control:	**0.00027**
shNT + LPS vs shNR4A3 + LPS:	**2.99E-05**

C.

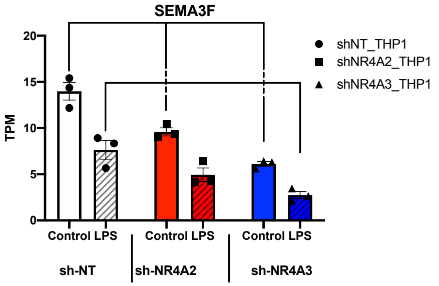

	Padj
shNT Control vs shNR4A2 Control:	**0.014**
shNT + LPS vs shNR4A2 + LPS:	0.081

	Padj
shNT Control vs shNR4A3 Control:	**8.87E-10**
shNT + LPS vs shNR4A3 + LPS:	**6.74E-08**
